# Genetic Modeling and Genomic Analyses of Yearling Temperament in American Angus Cattle and Its Relationship With Productive Efficiency and Resilience Traits

**DOI:** 10.3389/fgene.2022.794625

**Published:** 2022-04-04

**Authors:** Amanda B. Alvarenga, Hinayah R. Oliveira, Stephen P. Miller, Fabyano F. Silva, Luiz F. Brito

**Affiliations:** ^1^ Department of Animal Sciences, Purdue University, West Lafayette, IN, United States; ^2^ Centre for Genetic Improvement of Livestock, Department of Animal Biosciences, University of Guelph, Guelph, ON, Canada; ^3^ American Angus Association, Angus Genetics Inc., St Joseph, MO, United States; ^4^ Department of Animal Sciences, Federal University of Vicosa, Viçosa, Brazil

**Keywords:** behavior, genome-wide association study, livestock, long noncoding RNA genes, xlinked trait, weighted single-step GBLUP

## Abstract

Cattle temperament has been considered by farmers as a key breeding goal due to its relevance for cattlemen’s safety, animal welfare, resilience, and longevity and its association with many economically important traits (e.g., production and meat quality). The definition of proper statistical models, accurate variance component estimates, and knowledge on the genetic background of the indicator trait evaluated are of great importance for accurately predicting the genetic merit of breeding animals. Therefore, 266,029 American Angus cattle with yearling temperament records (1–6 score) were used to evaluate statistical models and estimate variance components; investigate the association of sex and farm management with temperament; assess the weighted correlation of estimated breeding values for temperament and productive, reproductive efficiency and resilience traits; and perform a weighted single-step genome-wide association analysis using 69,559 animals genotyped for 54,609 single-nucleotide polymorphisms. Sex and extrinsic factors were significantly associated with temperament, including conception type, age of dam, birth season, and additional animal–human interactions. Similar results were observed among models including only the direct additive genetic effect and when adding other maternal effects. Estimated heritability of temperament was equal to 0.39 on the liability scale. Favorable genetic correlations were observed between temperament and other relevant traits, including growth, feed efficiency, meat quality, and reproductive traits. The highest approximated genetic correlations were observed between temperament and growth traits (weaning weight, 0.28; yearling weight, 0.28). Altogether, we identified 11 genomic regions, located across nine chromosomes including BTAX, explaining 3.33% of the total additive genetic variance. The candidate genes identified were enriched in pathways related to vision, which could be associated with reception of stimulus and/or cognitive abilities. This study encompasses large and diverse phenotypic, genomic, and pedigree datasets of US Angus cattle. Yearling temperament is a highly heritable and polygenic trait that can be improved through genetic selection. Direct selection for temperament is not expected to result in unfavorable responses on other relevant traits due to the favorable or low genetic correlations observed. In summary, this study contributes to a better understanding of the impact of maternal effects, extrinsic factors, and various genomic regions associated with yearling temperament in North American Angus cattle.

## Introduction

Genetic evaluation schemes have been refined over time concomitantly with consumers, producers, and industry requirements. A recent American Angus Association (AAA) producers’ survey revealed docility (or temperament) as one of the top three traits to be prioritized in US Angus cattle breeding programs (Santos et al., 2019). Behavioral traits are important due to their impact on animal performance [e.g., growth (Hoppe et al., 2010)], meat quality (King et al., 2006; Coutinho et al., 2017; Sant’Anna et al., 2019), reproduction (Cooke et al., 2011; Valente et al., 2015; Cziszter et al., 2016), and immunity (Burdick et al., 2011). Other substantial influences include handler safety, animal welfare, longevity (Neja et al., 2015), efficiency of management systems, and economic profit (Carlstrom et al., 2014). For instance, Busby (2015) has reported an average profit of $46.63 per docile animal compared to $7.62 per animal with aggressive behavior. Temperament is one of a plethora of behavioral measurements, and it is defined as “the animal response to handling or forced movement by humans” (Burrow et al., 1988). Temperament can be assessed in multiple ways, including objective measurements [e.g., flight time (Burrow et al., 1988)] and subjective evaluations (Tulloh, 1961; Dickson et al., 1970; Kilgour, 1975).

A wide range of heritability estimates [average equal to 0.27, ranging from 0.03 to 0.67 (Sato, 1981; Hearnshaw and Morris, 1984; Hoppe et al., 2010; Rolfe et al., 2011)] has been reported for beef cattle temperament traits, supporting the idea that cattle temperament can be improved through genetic or genomic selection. Studies underlying the genetic background of temperament have also been performed (Esmailizadeh et al., 2008; Chan, 2012; Hanna et al., 2014; Riley et al., 2016; Garza-Brenner et al., 2017; Macleod et al., 2019; Costilla et al., 2020). In total, 29 quantitative trait loci (QTL) have been reported, in which 19 were identified for temperament in beef or dual-purpose cattle (Esmailizadeh et al., 2008; Glenske et al., 2011; Riley et al., 2016; Dos Santos et al., 2017; Garza-Brenner et al., 2017; Hu et al., 2019). Additionally, orthologous genes have been reported to be controlling behavioral indicator traits across various species, including humans (Costilla et al., 2020; Alvarenga et al., 2021). However, to the best of our knowledge, none of the mentioned studies have used large datasets [the maximum number of records in previous studies was 9,223 individuals (Costilla et al., 2020)] recorded in a broad array of geographic, environmental, and climatic zones, nor genetically evaluated temperament in the US Angus population, which is the most predominant breed in the largest producer of beef cattle in the world (Greenwood, 2021). Along with the breeding goals, environmental conditions, management practices, genetic population parameters (Hidalgo et al., 2020), and trait definitions change over time. Herein, routine updates of the statistical models and re-estimation of variance components are required, as the accuracy of estimated breeding values (EBVs) relies on properly defined genetic models and accurate genetic parameter estimates.

Therefore, our overarching goal was to better understand the variation of temperament at the yearling age across the US using a large dataset provided by the AAA through the Angus Genetics Inc. (AGI—American Angus Association; Saint Joseph, MO, US), from the non-genetic, genetic, and genomic aspects. The understanding of the non-genetic factors impacting temperament can guide the statistical modeling and provide a better understanding of nurture impacting temperament. Thereafter, up-to-date statistical models and variance components will allow more accurate EBV estimates and a more powerful genome-wide association to understand the genetic background and the biological mechanisms associated with cattle temperament. With that, the specific objectives of this study were to 1) identify the non-genetic effects (e.g., management events) influencing yearling temperament; 2) define an optimal statistical model (in terms of systematic and random effects) to be used in routine genetic and genomic evaluations of yearling temperament in North American Angus cattle; 3) estimate variance components for yearling temperament; 4) estimate approximated genetic correlations between yearling temperament and 20 productive efficiency and resilience traits, including calving ease, growth traits, carcass measurements, body conformation, susceptibility to high altitude disease, hair shedding score, and other mature cow measurements; and 5) identify genomic regions and candidate genes controlling yearling temperament in Angus cattle based on weighted single-step genome-wide association studies, followed by metabolic pathway (functional enrichment) analyses and a comparison with previously reported QTLs.

## Materials and Methods

### Data

All the data used in this study are from purebred and commercial Angus cattle registered in the AAA, born between 1990 and 2018, and recorded for temperament score at yearling age. Yearling temperament score is a subjective measurement (i.e., visually evaluated by the farmers/handlers), and it is recorded from 320 to 440 days of age (yearling) when the animal exits a chute, which is consistent within contemporary groups (Northcutt and Bowman, 2007). Temperament is scored on a one-to-six scale, in which a score of 1 represents a docile animal (i.e., desirable behavior) and a score of 6 represents a very aggressive (more temperamental) animal. A complete description of the scores and measurement guidelines used by the AAA are shown in Table 1.

**TABLE 1:** Description^a^ of yearling temperament scores and number of animals per level in American Angus cattle.

Score	Description^a^	N
1	Docile—mild disposition. Gentle and easily handled. Stands and moves slowly during processing. Exits chute calmly	191,402
2	Restless—quieter than average but may be stubborn during processing. May try to back out of chute. Some flicking of tail. Exits chute promptly	58,927
3	Nervous—typical temperament is manageable, but nervous and impatient. Displays a moderate amount of struggling, movement, and tail flicking. Exits chute briskly	13,615
4	Flighty (wild)—jumpy and out of control, quivers, and struggles violently. May bellow and froth at the mouth. Displays continuous tail flicking. Defecates and urinates during processing. May jump when penned individually. Exhibits long flight distance and exits chute wildly	1,778
5	Aggressive—may be similar to score 4, but added aggressive behavior, fearfulness, extreme agitation, and continuous movement, which may include jumping and bellowing while in the chute. Exits chute frantically and may exhibit attack behavior when handled alone	235
6	Very aggressive—thrashes about or attacks wildly when confined in small, tight places. Pronounced attack behavior	72

a Description and scoring guidelines from the ANGUS Journal Report, October 2007 (Northcutt and Bowman, 2007); N: Number of animals per score of yearling temperament after the data quality control.

Records from 675,678 Angus cattle with temperament score at yearling age were available. Few animals had scores of 4 (1,778 animals), 5 (425 animals), or 6 (151 animals). Therefore, these three categories were combined, which resulted in 83.8% of animals with score 1, 12.9% animals with score 2, 2.8% animals with score 3, and 0.5% animals with score 4+ (representing the scores 4, 5, and 6). A quality control procedure was performed to remove animals with missing information for the systematic effects evaluated and to warranty enough variability within their levels (i.e., at least three animals per level). Thereafter, the herds (where animals were located at birth, weaning, and yearling) exclusively represented by one score (e.g., all animals with score 1) were removed from the dataset. This criterion was used to avoid bias generated by the farmer/evaluator and allow variability within the levels. Additionally, data from animals younger than 320 days and older than 440 days at the time of temperament assessment were also removed. After quality control, there were 266,029 animals comprising 147,671 bulls, 3,332 steers, and 115,026 females with yearling temperament scores 1 (71.9%), 2 (22.2%), 3 (5.1%), or 4+ (0.8%). These remaining animals were born between 2001 and 2018. The number of animals per yearling temperament score after the quality control is shown in Table 1.

### Statistical Model Definition and Genetic Parameter Estimation

Before consideration of the statistical model to be used, several options were tested for environmental (systematic) and genetic (direct and maternal genetic) effects. The statistical models used in this study were defined based on two steps: 1) definition of the systematic effects and contemporary group (*CG*) and 2) definition of direct genetic, maternal genetic, and/or maternal environmental effects. Concomitantly to the second step, the genetic parameters were estimated.

The first step was performed using the *glm* function available in R software (R Core Team, 2019), considering a quasi-binomial family distribution. Stepwise (i.e., backward elimination) subroutines for selecting the best subset of covariates were used for the systematic model definition and to define the *CG*. The best fitted non-genetic model for yearling temperament rendered a coefficient of determination equal to 0.35. The model included age of dam (*AOD*; 10 levels), *CG* (22,322 levels), and conception type (*ET*; two levels: embryo transference or natural conception) as categorical systematic effects and calf age deviation from 365 days (*CALFDEV*) as a linear covariate. *AOD* was classified following the Beef Improvement Federation Guidelines [BIF (Cundiff et al., 2018)]. *CG* was defined as the concatenation of birth date (month-year), birth herd, weaning date (month-year), weaning herd, if the animal was submitted to a creep feeding system, if the animal had ultrasound information, and date (month-year), herd, sex, and group age deviation when yearling temperament was recorded.

After defining the systematic model and the optimal *CG*, four mixed animal models were tested by incorporating four random effects: *CG*, additive direct genetic, maternal genetic, and/or maternal environmental effects. The four models and their corresponding components are listed in Table 2.

**TABLE 2:** Random effects included in the four animal models for yearling temperament in American Angus cattle.

Models	Components				
	$\sigma_{CG}^{2}$	$\sigma_{u}^{2}$	$\sigma_{m}^{2}$	$\sigma_{u,m}$	$\sigma_{p}^{2}$
D	**✓**	**✓**	—	—	—
DMG	**✓**	**✓**	**✓**	**✓**	—
DMP	**✓**	**✓**	—	—	**✓**
DMGP	**✓**	**✓**	**✓**	**✓**	**✓**

D: reduced model fitting the additive direct genetic effect; DMG: model fitting the additive direct and maternal genetic effects; DMP: model fitting the additive direct genetic and maternal environmental effects; DMGP: complete model including additive direct genetic, maternal genetic, and maternal environmental effects; $\sigma_{CG}^{2}$ : contemporary group variance; $\sigma_{u}^{2}$ : additive direct genetic variance; $\sigma_{m}^{2}$ : maternal genetic variance; $\sigma_{u,m}$ : covariance between additive direct and maternal genetic effects; $\sigma_{p}^{2}$ : maternal environmental variance; **✓**: component was included in the model.

Bayesian threshold models based on the Gibbs sampler and Markov Chain Monte Carlo (MCMC) algorithm were used to estimate the genetic parameters and, thereafter, compare the models. thrgibbs1f90 software (Tsuruta and Misztal, 2006) was used with a chain length varying from 500,000 to 1,000,000 iterations, assuming a burn-in from 50% to 75%, and a thin of 50 or 100. Non-informative prior information based on the inverse-chi-squared distribution was assumed for variance components. The models are presented below. The reduced model is defined as follows:

D model:l=Xb+Ww+Zu+e [1]

where l is a vector of observations for the underlying threshold trait measured on the liability scale for yearling temperament score; b  is a vector of systematic effects, including *AOD* and *ET* and *CALFDEV* as a linear covariate, b  assumes a uniform distribution [non-informative prior, $b\;∼\;N\left(0,\Sigma_{b}⊗I\right)$ ]; **w** is a vector of CG random effects, *CG*, $w∼N\left(0,I\sigma_{w}^{2}\right)$ ; u is a vector of direct genetic effect, $u∼N\left(0,A\sigma_{u}^{2}\right)$ ; e is a residual vector, $e∼N\left(0,I\sigma_{e}^{2}\right)$ ; X , W , and Z are incidence matrices of b , ***w***, and  u , respectively. The $\Sigma_{b}$ is a diagonal matrix with large values to represent vague prior knowledge, A is the pedigree-based relationship matrix assuming 10 generations back to the animals with phenotypes, I is an identity matrix, and ⊗ denotes the Kronecker product. $\sigma_{w}^{2}$ , $\sigma_{u}^{2}$ , and $\sigma_{e}^{2}$ are the *CG*, additive genetic, and residual variances, respectively. The three models incorporating maternal effects are as follows:

$$DMG\;\mathrm{mod}\text{e}l:l=Xb+Ww+Zu+Z_{2}m+e$$ [2]

where u is a vector of direct genetic effect on the liability scale; m is the maternal genetic effect; $Z_{2}$ is an incidence matrix of m , $m|\sigma_{m}^{2}\;∼N\left(0,A\sigma_{m}^{2}\right)$ ; the covariance structure of genetic random effects ( u and m ) was defined as A⊗∑ , in which $\Sigma =\left[\begin{array}{cc} \sigma_{u}^{2} & \sigma_{u,m}\\ \sigma_{u,m} & \sigma_{m}^{2} \end{array}\right]$ ; $\sigma_{m}^{2}$ and $\sigma_{u,m}$ are the maternal genetic variance and direct and maternal genetic covariance, respectively. All other terms were previously defined in Eq. 1.

DMP model:l=Xb+Ww+Zu+Sp+e [3]

where p is a vector of maternal environmental effect, $p|\sigma_{p}^{2}\;∼\;N\left(0,I\sigma_{p}^{2}\right)$ ; S is an incidence matrix of p , and $\sigma_{p}^{2}$ is the maternal environmental variance. All other terms were previously defined in Eq. 1.

$$DMPG\;\mathrm{mod}\text{e}l:\;l=Xb+Ww+Zu+Z_{2}m+Sp+e$$ [4]

where all the components of the DMGP model were previously defined in Eqs 1–3. The systematic effects were the same for all four models.

A total of 819,303 animals (242,570 bulls, 298 steers, and 576,435 cows) born between 1928 and 2018 were included in the A matrix. Of those, 775,176 animals had both known parents, 35 animals had one known parent, and 44,092 animals had unknown parents. The MCMC convergence was verified using the Heidelberger and Welch (Heidelberger and Welch, 1983) and Geweke (Geweke, 1991) criteria, both implemented in the *boa* package (Smith, 2007) of R software (R Core Team, 2019). The variance components and heritability estimates were obtained using postgibbsf90 software (Tsuruta and Misztal, 2006). The direct ( $h_{d}^{2}$ ), maternal ( $h_{m}^{2}$ ), and total ( $h_{t}^{2}$ ) heritability estimates were computed, respectively, as follows:

$$h_{d}^{2}=\frac{\sigma_{u}^{2}}{\sigma_{t}^{2}}$$ [5]

$$h_{m}^{2}=\frac{\sigma_{m}^{2}}{\sigma_{t}^{2}}$$ [6]

$$h_{t}^{2}=\frac{\sigma_{u}^{2}+1.5\sigma_{u,m}+\;0.5\sigma_{m}^{2}}{\sigma_{t}^{2}}$$ [7]

where $\sigma_{t}^{2}$ is the total variance (which was differently calculated for each model; i.e., $\sigma_{t}^{2}$ is equal to the sum of the $\sigma_{u}^{2}$ , $\sigma_{w}^{2}$ , and $\sigma_{e}^{2}$ elements for the D model; the sum of $\sigma_{u}^{2}$ , $\sigma_{w}^{2}$ , $\sigma_{u,m}$ , $\sigma_{m}^{2}$ , and $\sigma_{e}^{2}$ for the DMG model; the sum of $\sigma_{u}^{2}$ , $\sigma_{w}^{2}$ , $\sigma_{p}^{2}$ , and $\sigma_{e}^{2}$ for the DMP model; and the sum of $\sigma_{u}^{2}$ , $\sigma_{w}^{2}$ , $\sigma_{u,m}$ , $\sigma_{m}^{2}$ , $\;\sigma_{p}^{2}$ , and $\sigma_{e}^{2}$ for the DMGP model).

The models were compared based on the linear regression method (Legarra and Reverter, 2018), which estimates bias, dispersion, and accuracies comparing two subsets of breeding values (EBVs) estimated using a partial and whole dataset. The whole dataset comprises all animals with temperament records (i.e., 266,029). The partial dataset was defined as animals in the whole dataset but masking the phenotype of the animals born in 2018 (13,202 animals), which was defined as the validation group. The EBVs from the partial and whole datasets were obtained fixing the variance components in thrgibbs1f90 with 10,000 iterations, 1,000 burn-in, and 5 thin. Bias, dispersion, and predictive accuracies were calculated for the validation animals.

### Effect of Sex and Extrinsic Variables on Yearling Temperament

Sex and extrinsic factors available in the AAA dataset were submitted to a pairwise comparison using the *lsmeans* package (Lenth, 2016) implemented in R software (R Core Team, 2019). A linear model including calf age deviation from 365 days as a linear covariate and the following target independent variables as a categorical fixed effect, such as age of dam, conception type (embryo transfer or natural conception), parity type (single or twin), sex, birth season, if the animal was under a creep-feeding system, and if the animal had ultrasound and feed intake information, was used. In other words, the mean presented for each level of the fixed effect was adjusted for the remaining factors.

### Approximated Genetic Correlations

The genetic relationship between yearling temperament and other productive efficiency and resilience traits is of great importance for the design of breeding programs and development of selection indexes. Due to limited access to the raw datasets and computational limitations, the genetic correlation between yearling temperament and 20 other relevant traits (Table 3) was assessed based on the correlation between the EBVs. The EBVs for yearling temperament were from the *Statistical Model Definition and Genetic Parameter Estimation* while fixing the variance components in thrgibbs1f90. The EBVs for the other traits were from the official genetic evaluation performed by the AAA, which includes the following: calving ease [calving ease direct (CED) and maternal calving ease (CEM)], body weight [birth weight (BW), weaning weight (WW), and yearling weight (YW)], residual average daily gain (RADG), dry-matter intake (DMI), yearling height (YH), scrotal circumference (SC), carcass and meat quality measurements [carcass weight (CW), marbling score (MARB), ribeye area (REA), and fat thickness (FAT)], conformation [foot angle (FOOT)], adaptation [high altitude disease susceptibility—pulmonary artery pressure (PAP) and hair shedding score (HS)], and other mature cow measurements [fertility—heifer pregnancy (HP), mature weight (MW), mature height (MH), and maternal milk (MILK)]. A complete description of the traits is shown in Table 3. Data from animals with EBV accuracies lower than 0.25 for both evaluated traits were disregarded for the correlation. A weighted Pearson correlation ( $r_{w1,2}$ ) between EBV pairs was calculated. The calculation of $r_{w1,2}$ comprised five steps:

**TABLE 3:** Description of production traits evaluated to be genetically correlated with yearling temperament score, as defined by the American Angus Association.

Symbol	Trait EBV	Unit and description^a^
CED	Calving ease-direct	Percentage of unassisted births (calf measurement/direct genetic effect)
BW	Birth weight	Pounds
WW	Weaning weight	Pounds, direct genetic effect
YW	Yearling weight	Pounds
RADG	Residual average daily gain	Pounds per day, the sire’s ability for post-weaning gain in his progeny given a constant amount of feed consumed
DMI	Dry-matter intake	Pounds per day
YH	Yearling height	Inches
SC	Scrotal circumference	Centimeters
PAP	Pulmonary artery pressure	Probability, the sire’s ability to produce a progeny with lower (or greater) pulmonary arterial pressures probability, decreasing (or increasing) the risk of contracting high altitude diseases
HP	Heifer pregnancy	Percentage, it measures the ability of the sire’s daughters to become pregnant as first-calf heifers during a normal breeding season
CEM	Calving ease maternal	Percentage of unassisted births (cow measurement/maternal genetic effect)
MILK	Maternal milk	Pounds of calf weaned, the sire’s genetic merit for milk and mothering ability as expressed in his daughters (maternal genetic effect of WW)
MW	Mature weight	Pounds
MH	Mature height	Inches
CW	Carcass weight	Pounds
MARB	Marbling score	Marbling score
RE	Ribeye area	Square inches
FAT	Fat thickness	Inches, the sire’s ability to transmit fat thickness at the 12th rib (as measured between the 12th and 13th ribs) to his progeny
FOOT	Foot angle score	Foot-angle score, the sire’s ability to transmit ideal foot angle to his progeny, of which a lower value is desirable
HS	Hair shed score	Hair shed score, the sire’s ability to transmit early (or late) summer hair shedding, of which a lower value is desirable

EBV: Estimated breeding value.

a Source: American Angus Association website (www.angus.org/mobile/nce/definitions.aspx).

**1** ^**st**^ **.** Calculation of the weights ( $w_{i}$ ), which was based on the accuracies of the respective traits to be correlated for the animal *i*:

$$w_{i}=\frac{REL_{1i}*REL_{2i}}{\sqrt{REL_{1i}*REL_{2i}}}$$ [8]

where $w_{i}$ is the weighting for the animal *i*; $REL_{1i}$ and $REL_{2i}$ are the squared accuracies of the EBV for traits 1 and 2 for animal *i*, respectively. The approximate squared accuracies ( $REL_{1}$ and $REL_{2}$ ) of the EBV was calculated using accf90GS software (Misztal et al., 2013).

**2** ^**nd**^ **.** Calculation of the weighted mean for traits 1 and 2:

$$m_{1}=\;\frac{\sum_{i=1}^{n}\left(EBV_{1i}*w_{i}\right)}{\sum_{i=1}^{n}w_{i}}\;\;\;\;\;and\;\;\;\;\;m_{2}=\;\frac{\sum_{i=1}^{n}\left(EBV_{2i}*w_{i}\right)}{\sum_{i=1}^{n}w_{i}}$$ [9]

where $m_{1}$ and $m_{2}$ are the weighted mean for traits 1 and 2; $EBV_{1i}$ and $EBV_{2i}$ are the EBVs for traits 1 and 2, respectively, both measured in animal *i*, and *n* is the total number of animals. The EBV for yearling temperament was converted to the probability of the animal being docile (score 1), in which greater EBVs represent higher probability of the animal receiving a score of 1 (docile).

**3** ^**rd**^ **.** Calculation of the weighted variances:

$$s_{1}=\;\frac{\sum_{i=1}^{n}w_{i}{\left(EBV_{1i}-m_{1}\right)}^{2}}{\sum_{i=1}^{n}w_{i}}\;\;\;\;\;and\;\;\;\;\;s_{2}=\;\frac{\sum_{i=1}^{n}w_{i}{\left(EBV_{2i}-m_{2}\right)}^{2}}{\sum_{i=1}^{n}w_{i}}$$ [10]

where $s_{1}$ and $s_{2}$ are the weighted variance for traits 1 and 2 and all the remaining parameters have been previously described.

**4** ^**th**^ **.** Calculation of the weighted covariance:

$$s_{1,2}=\;\frac{\sum_{i=1}^{n}w_{i}\left(EBV_{1i}-m_{1}\right)\left(EBV_{2i}-m_{2}\right)}{\sum_{i=1}^{n}w_{i}}$$ [11]

where $s_{1,2}$ is the weighted covariance between EBVs for traits 1 and 2 and all the remaining parameters have been previously described.

**5** ^**th**^ **.** Calculation of weighted correlation:

$$r_{w1,2}=\;\frac{s_{1,2}}{\sqrt{s_{1}*s_{2}}}$$ [12]

where $r_{w1,2}$ is the weighted correlation between EBVs for traits 1 and 2 and all the remaining parameters have been previously described.

Finally, the standard errors (± SE) of the genetic correlation metric (i.e., $r_{w1,2}$ ) were calculated as follows:

$$SE=\sqrt{\frac{1-r_{w1,2}}{n-2}}$$ [13]

where all the parameters have been described. The 95% confidence intervals were obtained as mean ± 1.96 × SE (Altman and Bland, 2005).

### Weighted Single-Step Genome-Wide Association Analyses

A weighted single-step genome-wide association study [WssGWAS (Wang et al., 2012)] was performed to identify candidate regions controlling yearling temperament. From the 266,029 animals with phenotypic information for yearling temperament, 69,559 were genotyped. Animals were genotyped with various SNP arrays as part of ongoing commercial genotyping activities by breeders for genetic evaluation purposes, resulting in a market set of 54,609 SNPs. Commercial genotyping products were from Zoetis, including i50K (www.zoetisus.com/animal-genetics/media/documents/i50k-00001_50k-sellsheet.pdf) and HD50K (www.zoetisus.com/animal-genetics/beef/hd-50k/hd-50k-for-black-angus.aspx), and Neogen GeneSeek, including GeneSeek Genomic Profile Low-Density (GGPLD; 40 K SNPs), High-Density (GGPHD; 80 K SNPs), GGPUHD (150 K SNPs), and AngusGS. Both Zoetis and Neogen provided, for genomic evaluation purposes, an imputed (average and standard deviation imputation accuracy equal to 99.72% and 0.87) SNP set similar to the Illumina BovineSNP50V2 and Illumina BovineSNP50V3 (Illumina, Inc., San Diego, CA), respectively, and mapped to the bovine genome assembly UMD3.1. However, the genomic coordinates were converted to ARS-UCD1.2 bovine genome assembly (Medrano, 2017; Rosen et al., 2018) using the *biomaRt* R package (Durinck et al., 2009).

Quality control (QC) procedures were applied to remove genotyped individuals with a call rate lower than 90% and pedigree errors. SNP genotypes with a call rate lower than 90%, an MAF lower than 0.01, and a deviance of heterozygous genotype from the Hardy Weinberg Equilibrium higher than 0.15 were also removed. Both autosomal chromosomes and pseudo-autosomal regions of the X chromosome were used in the WssGWAS. SNPs located on approximated pseudo-autosomal regions [PAR; SNP with a genomic coordinate above X:133,300,518 bp; 109 SNPs; (Johnson et al., 2019)] represented the X chromosome because it follows an autosomal-chromosome inherence and, therefore, were analyzed as autosomal SNPs (Johnson et al., 2019). preGSf90 software (Aguilar et al., 2014) was used to perform the QC, in which 69,441 genotyped animals and 42,662 SNPs remained for further analyses. As a WssGWAS was performed, the pedigree-based relationship matrix ( A ) and G were combined into the H matrix (Misztal et al., 2009; Aguilar et al., 2010; Christensen and Lund, 2010; Wang et al., 2014). The H inverse $\left(H^{-1}\right)$ is defined as follows:

$$H^{-1}=A^{-1}+\left[\begin{array}{cc} 0 & 0\\ 0 & {\left(0.90G+0.10A_{22}\right)}^{-1}-A_{22}^{-1} \end{array}\right]$$ [14]

where $A_{22}$ is a pedigree-based relationship matrix for the genotyped animals. The pedigree dataset contained animals traced back up to four generations, resulting in 578,821 animals. The **G** matrix was constructed as in the study by VanRaden (2008):

G=RDR′ [15]

where **R** is a matrix of gene content adjusted for observed allele frequencies and **D** is a diagonal matrix of SNP weights. The weights were derived from the third iteration’s SNP solutions using a nonlinear approach described by VanRaden (2008) and Cole et al. (2009).

The optimal or selected statistical model was used to perform the WssGWAS. The vector of direct genetic effects was assumed to follow a normal distribution, $u|\sigma_{u}^{2}∼N\left(0,H\sigma_{u}^{2}\right)$ . The variance components were fixed based on the estimates given by the pedigree-based genetic analyses. thrgibbs1f90 software (Tsuruta and Misztal, 2006) with a 10,000 chain length, 1,000 burn-in, and 5 thin was used to calculate the genomic estimated breeding values (GEBVs). The SNP effects were obtained by back-solving the GEBVs using postGSf90 software (Stranden and Garrick, 2009; Wang et al., 2012; Aguilar et al., 2014). Sliding genomic windows of five SNPs were considered to calculate the effect of a certain genomic region on yearling temperament and the proportion of the total additive genetic variance explained. The window size was defined based on linkage disequilibrium (LD) decay in Angus cattle [LD higher than 0.19 (Lu et al., 2012)]. Genomic windows explaining more than 0.20% of the total additive genetic variance were considered as relevant and subjected to further investigations.

The genomic coordinates were based on the ARS-UCD1.2 bovine genome assembly (Medrano, 2017; Rosen et al., 2018). Quantitative trait loci (QTL) curated in the Cattle QTL DataBase [Cattle QTLdb (Hu et al., 2019); www.animalgenome.org; access date: September 27, 2021] and located within the selected genomic windows were identified. Gene annotation information was retrieved from Ensembl using the *biomaRt* R package (Durinck et al., 2009). Functional annotation was performed in terms of Gene Ontology (GO) biological processes [GO_BP; (Blake et al., 2013)] and metabolic pathways of the Kyoto Encyclopedia of Genes and Genomes [KEGG; (Kanehisa and Goto, 2000)] available in the DAVID database [david.ncifcrf.gov/tools.jsp; (Dennis et al., 2003); access date: September 27, 2021].

## Results

### Sex and Extrinsic Variables Affecting Yearling Temperament

Yearling temperament was significantly associated with sex and extrinsic factors, which includes reproductive conception type, age of dam, birth season, creep-fed or non–creep-fed animals, and if there is ultrasound and/or feed intake recording (Supplementary Table S1). Parity type (i.e., single or twin) was the only factor not significantly associated with temperament.

**FIGURE 1:**
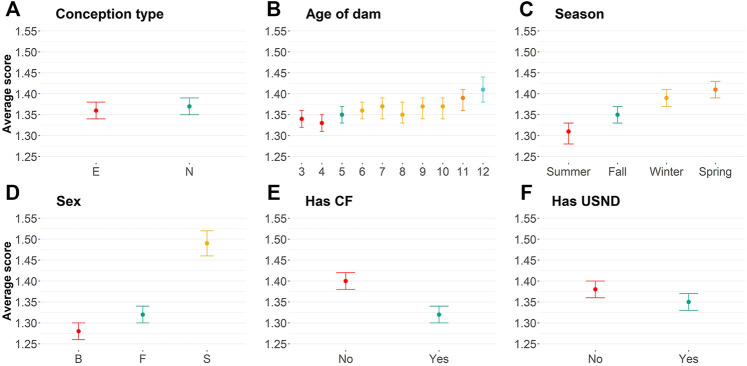
Average of temperament score using least-square means for **(A)** conception type [naturally conceived (N) or embryo transference (E)]; **(B)** Age of the dam in years; **(C)**: birth season; **(D)**: sex [bull (B), steers (S), and female (F)]; **(E)** if the animal was creep-fed; and **(F)**: if the animal had ultrasound information.

Embryo-transferred animals were statistically more docile (average equal to 1.36) than naturally conceived animals (average equal to 1.37; Supplementary Table S1; Figure 1A). Small differences in the averages are probably due to a skewed distribution toward docile scores (Table 1). The age of dam categorized by years (from three to 12 years of age) also influenced yearling temperament (Figure 1B; Table 2; Supplementary Table S1). Dams up to 4 years old raised more docile progenies (average equal to 1.34) than older dams (average equal to 1.41).

Birth season was statistically associated with yearling temperament score (*p* ≤ 0.05; Supplementary Table S1; Figure 1C). Animals that were born during the summer, fall, winter, and spring tend to range from docile to aggressive, respectively. The sex of the individual also influenced yearling temperament average. Bulls, females, and steers tended to be from more docile to more aggressive animals (*p* < 0.05; Supplementary Table S1; Figure 1D), respectively. Bulls had an average of yearling temperament score equal to 1.28, females had a temperament average equal to 1.32, and steers had a 1.49 average.

Our phenotypic analyses have also shown a positive relationship between implementation of the creep feeding system and animal temperament (Supplementary Table S1; Figure 1E). In short, creep-fed calves tend to be more docile (average of 1.32) than non–creep-fed calves (average of 1.40). Finally, animals that, in general, had additional recorded phenotypic collections were more docile (*p* ≤ 0.05; Supplementary Table S1; Figure 1F). For instance, animals that had ultrasound information tended to be more docile (average of 1.35) than animals without this measurement (average of 1.38). The same pattern was observed for feed intake information, which was expected because 90% of animals that had feed intake also had ultrasound information (i.e., 1,362 had ultrasound and feed intake information out of 1,513 animals, while the rest had only feed intake information).

### Model Choice and Parameter Estimation

We tested four animal models, which included direct genetic and maternal effects (Table 2). The results of the model incorporating both maternal effects (DMGP model including direct genetic, maternal genetic, and maternal environmental effects) are not presented because it did not converge even using up to 1 million iterations. The fitness measures of the models and genetic parameters are shown in Table 4. The DMG model, including direct and maternal genetic effect, provided a slight lower residual variance ( $\sigma_{e}^{2}$ = 0.19), which is beneficial, compared to the other models ( $\sigma_{e}^{2}$ = 0.21). The bias and dispersion from the forward validation were low for all models, varying from −0.02 to 0.02 and 0.95 and 0.97, respectively. Bias close to zero and dispersion close to one are desirable. Prediction accuracies were also similar across models, varying from 0.18 to 0.21. The computing time needed to obtain EBVs using the whole dataset was 136.2, 239.5, and 240.4 minutes for the D, DMP, and DMG models, respectively.

**TABLE 4:** Fitness of the model and genetic parameters for all models tested.

Model	$\sigma_{e}^{2}$	${\hat{\Delta }}_{v}$	${\hat{b}}_{v}$	${\hat{acc}}_{v}$	$h_{d}^{2}$	$h_{m}^{2}$	$h_{t}^{2}$	$r_{d,m}$
D	0.21 (0.02)	0.02	0.97	0.20	0.39 (0.01)			
DMG	0.19 (0.02)	0.00	0.95	0.18	0.44 (0.02)	0.04 (0.01)	0.38 (0.01)	−0.40 (0.04)
DMP	0.21 (0.02)	−0.02	0.96	0.21	0.38 (0.01)			

D: reduced model, including direct genetic effect; DMG: model including direct genetic and maternal genetic effects; DMP: model including direct genetic and maternal permanent environmental effects; $\sigma_{e}^{2}$ : residual variance (standard error)**;** ${\hat{\Delta }}_{v}$ : bias; ${\hat{b}}_{v}$ : dispersion; ${\hat{acc}}_{v}$ : predictive accuracy; $h_{d}^{2}$ : direct genetic heritability (standard error); $h_{m}^{2}$ : maternal genetic heritability (standard error); $r_{d,m}$ : correlation between direct-genetic and maternal (standard error). The variance components are on the liability scale.

Total yearling temperament heritability estimates ranged from 0.38 (DMP model) to 0.39 (D model) on the liability scale. Maternal genetic effects contributed to 4% of the total variation in yearling temperament on the liability scale (Table 4). Additionally, a strong negative genetic correlation was observed between direct and maternal genetic effects (−0.40; Table 4).

### Approximated Genetic Correlation Between Yearling Temperament and Other Relevant Traits

**FIGURE 2:**
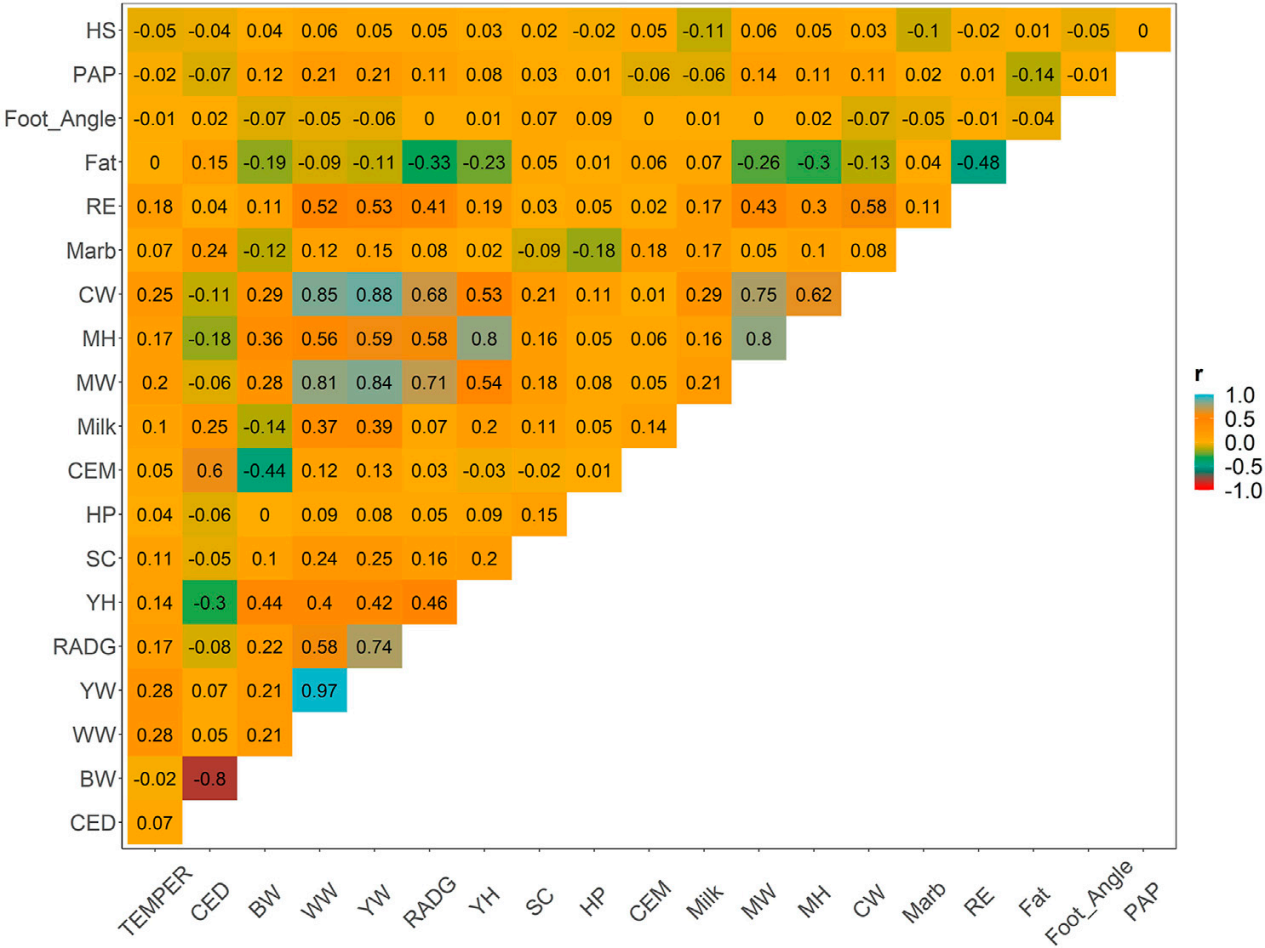
Weighted Pearson correlation among all estimated breeding values for relevant traits in the beef cattle industry.

Weighted correlations between the EBVs of yearling temperament and other key traits is shown in Figure 2. Additional correlations between all pairs of traits are provided for comparison. Other metrics, including the number of animals, and descriptive statistics (average, minimum, and maximum) of EBVs’ theoretical accuracy are shown in Supplementary Table S3. Low-to-moderate genetic correlation was observed between temperament and all other relevant traits, which varied from −0.05 (yearling temperament vs. hair shedding) to 0.28 (yearling temperament vs. weaning or yearling weight). Other approximated genetic correlation between pairs of traits was similar to the genetic correlation estimated using multiple-trait models used by the AAA (please see www.angus.org/Nce/Heritabilities).

Positive and favorable association between yearling temperament and growth traits (i.e., direct weaning, yearling, and mature weight; correlation from 0.20 to 0.28), feed efficiency (i.e., RADG; 0.17), precocity (i.e., scrotal score; 0.11), and carcass traits (i.e., carcass weight, ribeye by area; varying from 0.18 to 0.25) were observed. The remaining traits had low genetic correlations, varying from −0.05 (i.e., hair shedding) to 0.10 (i.e., maternal weaning weight).

### Weighted Single-Step Genome-Wide Association Analyses

The GEBVs from the D model were back-solved to generate the SNP window effects and the percentage of the total additive genetic variance explained by them. The WssGWAS was performed using 266,029 animals with phenotype, 69,441 animals with genotype for 42,662 SNPs located on autosomal and X chromosomes, and a pedigree containing 578,821 animals. The distribution of MAF is presented in Supplementary Figure S1, which was equally distributed across MAF intervals of 0.05.

**FIGURE 3:**
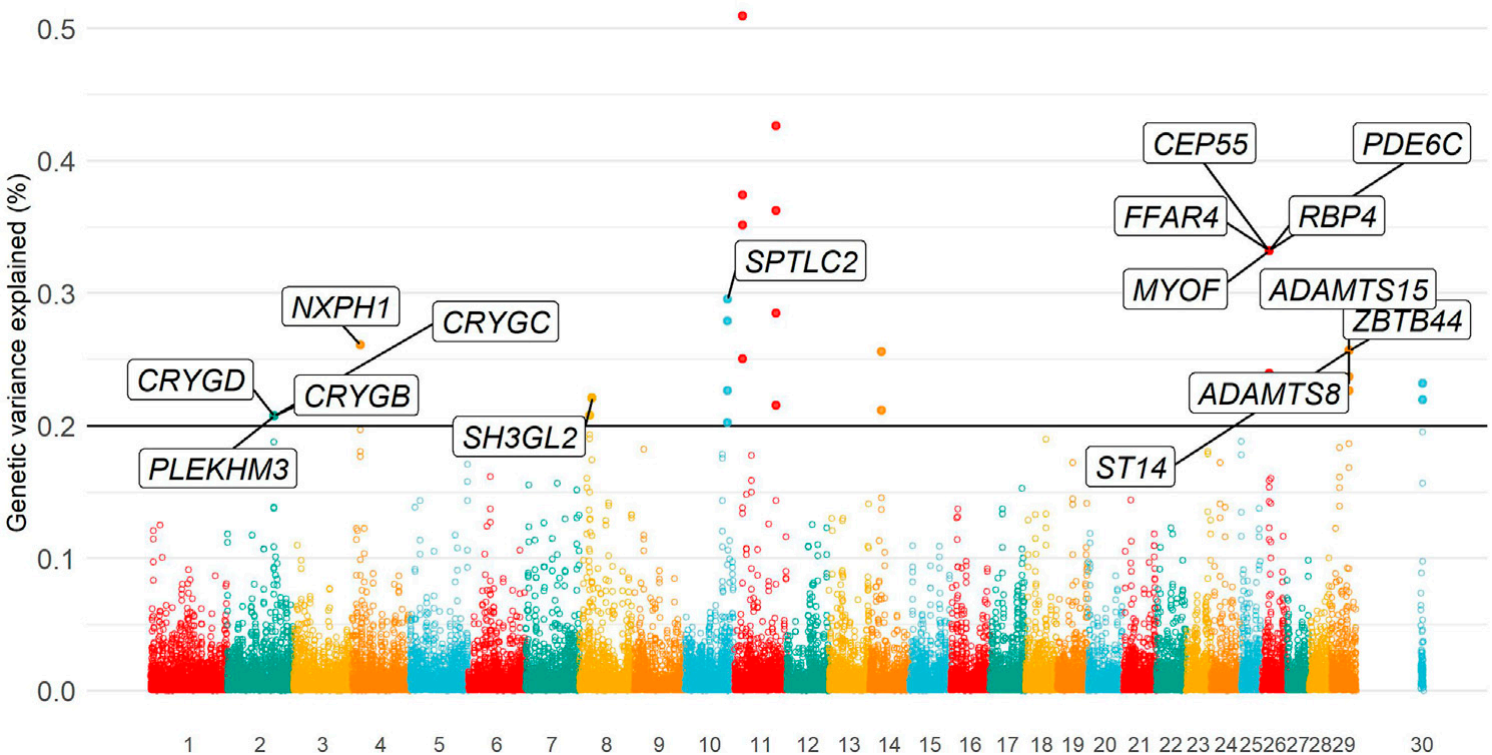
Manhattan plot of additive genetic variance explained by genomic windows for yearling temperament using the D model.

Eleven independent genomic regions (i.e., non-overlapping regions of five SNPs) were identified, in which each genomic region explained more than 0.20% of the total additive genetic variance. All genomic regions together explained 3.33% of the total additive genetic variance for temperament. Relevant genomic regions were located on BTA2, BTA4, BTA8, BTA10, BTA11, BTA14, BTA26, BTA29, and X chromosomes (Supplementary Table S4). The Manhattan plot of the additive genetic variance explained by genomic windows for yearling temperament is shown in Figure 3.

Twenty-five positional candidate genes were identified surrounding the 11 genomic windows, in which 18 genes were annotated and their biotype was classified as protein coding (17 genes) and noncoding RNA genes (one gene; Table 5; Supplementary Table S4). The two regions with the largest variance explained (i.e., 0.50%) are located on BTA11, and no annotated gene has been observed in that region. However, one of the regions had one gene categorized as long noncoding RNA (lncRNA; Supplementary Table S4). The richest genomic regions for identified genes were BTA2, BTA26, and BTA29. The main genes annotated in these regions are *PLEKHM3*, *CRYGD*, *CRYGC*, *CRYGB*, *RBP4*, *MYOF*, *CEP55*, *FFAR4*, *PDE6C*, *ST14*, *ZBTB44*, *ADAMTS8*, and *ADAMTS15.*

**TABLE 5:** Sample of the top genomic regions, the genes, and biological processes involved in these regions.

Gene name	Gene ensembl ID	CHR: start-end position	VE	Term
*CRYGB*	ENSBTAG00000048646	11:85006812-85223963	0.49	—
	ENSBTAG00000021770	2: 96181032-96426927	0.21	Eye lens protein
				Structural constituent of eye lens
*CRYGC*	ENSBTAG00000014783			Eye lens protein
				Methylation
				Visual perception
				Structural constituent of eye lens
*CRYGD*	ENSBTAG00000015054			Eye lens protein
				Visual perception
				Structural constituent of eye lens
*PDE6C*	ENSBTAG00000000445	26:14769909-14960555	0.33	Methylation
				Visual perception
*U6*	ENSBTAG00000042797	8: 26576536-26696264	0.22	—

CHR: Chromosome, VE: additive genetic variance explained by five-SNP window size (percentage).

**FIGURE 4:**
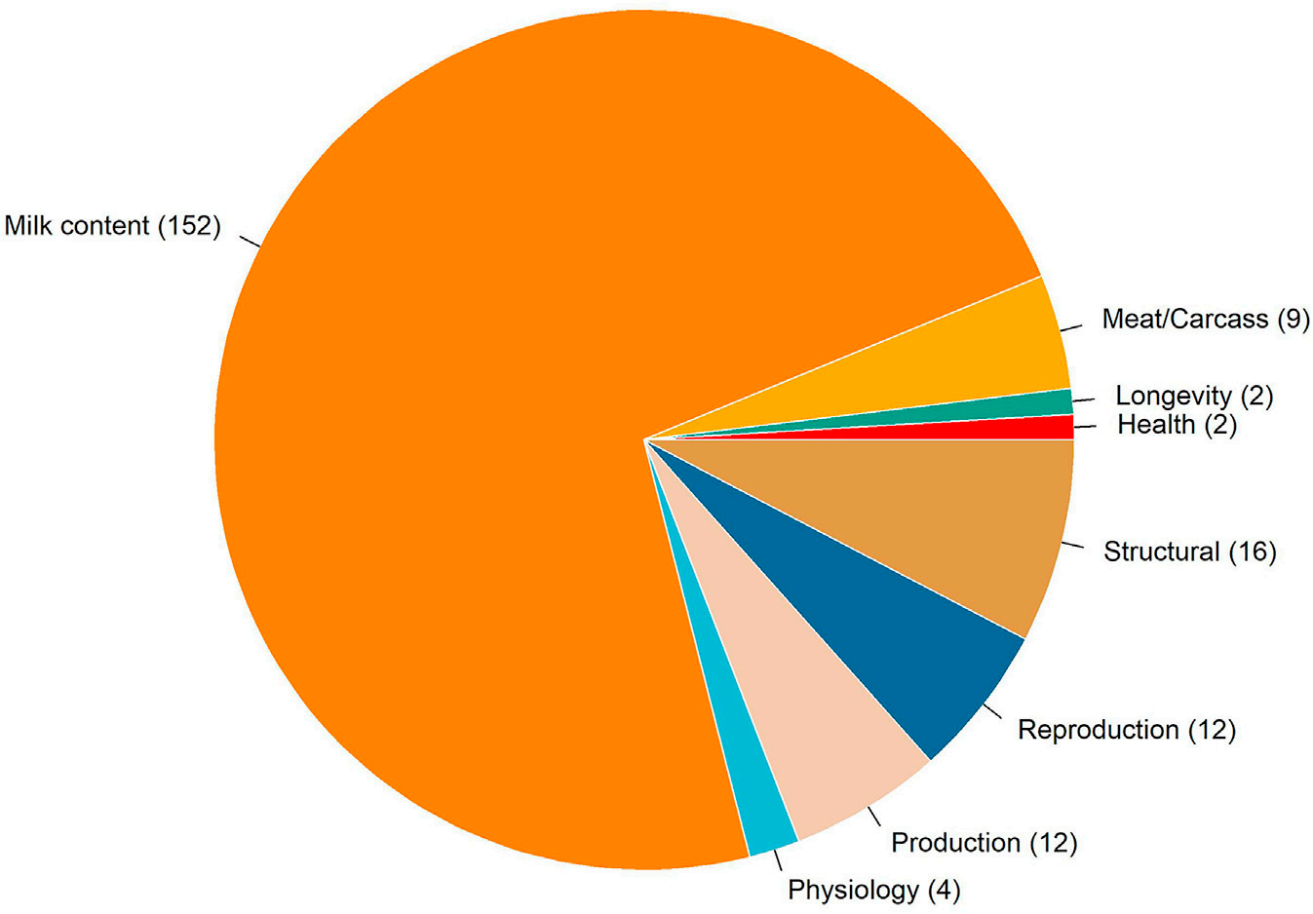
Chart of the trait-types associated with the quantitative trait loci overlapping with the genomic regions associated with temperament.

There were 210 QTLs annotated in an overlap with the genomic windows associated with temperament. Figure 4 shows the distribution of trait-type that these QTLs were previously associated with. In total, 72% of QTLs were previously associated with milk content, followed by 8% with structural problems, 6% with reproduction and production, and 4% with meat and carcass traits. Two QTLs for milking speed are located on a BTA14 genomic region explaining 0.26% of the total genetic variation. One gene overlapped in this region: *TOX* gene (thymocyte selection associated high mobility group box). In addition, two overlapped QTLs were annotated for longevity on BTA2, where four genes were identified, including three from the crystalline gamma family (i.e., *CRYGB, CRYGC*, and *CRYGD*) and *PLEKHM3* (pleckstrin homology domain containing M3).

To better understand the functions of the genes identified, we performed a functional analysis in DAVID, in which one GO biological process, one GO molecular function, and one term were significantly enriched based on the Benjamini test (*p* < 0.10). All terms were associated with vision processes. Visual perception (GO:0007601) was enriched by three genes located on BTA2, including *PDE6C* (phosphodiesterase 6C), *CRYGD*, and *CRYGC*. The last two genes from the *CRYG* family were also enriched in the structural constituent of eye lens (GO:0005212) and eye lens protein terms, in addition to a third CRYG family gene: *CRYGB* (Supplementary Table S5). Finally, another interesting process that those genes also participate in is methylation (Supplementary Table S5). In Table 5 is presented the genes enriched in the GO terms, as well as the genes located in the top regions.

## Discussion

### Sex and Extrinsic Variables Affecting Yearling Temperament

Pointing out extrinsic factors influencing yearling temperament may inform future management studies and, hence, improve management strategies implemented in herds as well as handlers’ safety. The elucidation of levels within the extrinsic factors intermeddling behavioral responses is also paramount to define and fit appropriate effects in the models, which can directly impact the prediction of EBVs and marker effects. Therefore, we have reported the connectivity between yearling temperament and farm experiences that the animal was subjected to, for which we used one of the largest beef cattle datasets available worldwide.

The statistical differences observed for embryo-transferred animals are potentially due to the genetic superiority of donors for many economically important traits, including temperament. An alternative hypothesis is associated with maternal ability and behavior during rearing. Particularly, foster/recipient cows could be (directly or indirectly) selected based on their maternal ability and easy-to-handle characteristics (Busby, 2015) in order to rear the biological progeny from high–genetic merit animals, and therefore, the behavior of these foster calves could be influenced by their foster mother characteristics.

Still, at the maternal level, younger dams raised more docile progenies than older dams. Similar trends were observed in Nellore cattle, where the average of temperament score tended to increase (i.e., being more aggressive) as the dam gets older (Barrozo et al., 2012). Studies have reported the high cognitive abilities of cattle, in which animals tend to avoid certain environments based on previous negative experiences (Ede et al., 2019). Thereupon, calves could reflect upon the behavior of their dams, in which an older cow, through memory acquisition, can become more aggressive or fearful across the years due to previous negative experiences (Kasimanickam et al., 2018).

In general, bulls have more dominant and aggressive behaviors than females (Reinhardt et al., 1987). Controversially, in recent studies, female beef cattle had been classified as more temperamental (Gauly et al., 2001). In this study, female Angus were on average more temperamental than male Angus (*p* < 0.05).

The birth-seasonal animal temperament pattern hinges on climate, grazing conditions, production system, and, consequently, on the farm location (McBride and Mathews, 2011; USDA–APHIS, United States Department of Agriculture-Animal and Plant Health Inspection Service, 2017). For instance, a study conducted by USDA–APHIS, United States Department of Agriculture-Animal and Plant Health Inspection Service, 2017 reported higher percentages of calves born during the spring in the Central US (78.4% of the animals) and West (64.0% of the animals) regions compared to calves born in the East region (43.0%). Additionally, the same study reported that over one-fourth of calves (27.9%) were born during the fall in the East region of the US, while only 15.3% and 7.8% were born in the West and Central regions, respectively (USDA–APHIS, United States Department of Agriculture-Animal and Plant Health Inspection Service, 2017). In the same report, a small proportion of cows calved during the summer [2.7% (USDA–APHIS, United States Department of Agriculture-Animal and Plant Health Inspection Service, 2017)]. Ultimately, the pattern of calves born per season, as presented by the USDA–APHIS, United States Department of Agriculture-Animal and Plant Health Inspection Service, 2017, is in agreement with our findings (10% summer, 18% fall, 30% spring, and 42% winter; Supplementary Table S1). Therefore, the season might reflect the region where most calves were born. In other words, animals born during the fall, winter, and spring mainly represent animals from the East, North, and Central-West regions, respectively. Another theory has to do with the size of the herd and/or contemporary group. Animals born during low season probably have few lot-mates and, as a consequence, lower probability of stressful social interactions.

Animal behavior has been associated with memory ability, in which positive and frequent human–animal interaction could positively impact future behavioral responses (Boivin et al., 2009). The creep feeding system and additional phenotypic measurement (e.g., ultrasound information) are generally associated with additional interaction with humans (Broadhead et al., 2018). A temperament study in Angus cows in Bulgaria reported that more docile cows have frequent contact with people (Karamfilov, 2022). Our phenotypic analyses showed a positive relationship between the use of creep feeding and if the animal had additional measurements (i.e., ultrasound and feed intake). On the other hand, an experimental study reported that despite efforts to give positive animal–human experiences during the handling, cattle would still be averse to the handling process, and its fear would increase (Petherick et al., 2002).

These findings provide a framework for understanding how sex and extrinsic factors influence temperament at the phenotypic level. Furthermore, it can provide insights to improve farm management to guide future epigenetic experimental studies. Environmental conditions regulating gene expression are well known for many traits, including docility (Cantrell et al., 2019). Hence, the listed environmental factors affecting yearling temperament can also be indicative of a programmer of epigenetics modifications.

### Model Choice and Parameter Estimation

The ultimate goal of model definition is to facilitate genetic improvement through more accurate estimation of variance components and breeding values (Bijma, 2006). Therefore, four animal models were tested in this study, including direct genetic and maternal effects (Tables 2, 4). Genetic parameters for the DMGP model, which includes direct genetic, maternal genetic, and maternal environmental effects, did not converge, which might be due to inherently problematic estimation of maternal effects (Meyer, 1994). The small average number of progenies per dam and grand-dam in our dataset represents an additional challenge for the estimation of maternal effects [i.e., average of 1.6 ± 1.82 progenies per dams (484,322 dams in the pedigree), 2.2 ± 4.9 grandchildren per grand-dam (total of 338,628 grand-dams in the pedigree), and 2.9 ± 10.2 great-grandchildren per great-grand-dam (total of 250,119 great-grand-dams in the pedigree)].

Among the converged models, the DMG model (i.e., including direct and maternal genetic effects) provided a slightly lower residual variance, suggesting the components are better capturing the total variance. Beckman et al. (2007) have also observed a positive effect of maternal effects (i.e., genetic and environment) on temperament in Limousin cattle. On the other hand, the other models presented slightly desirable dispersion and accuracy. Nevertheless, small differences were observed among the models considering the genetic parameters and linear regression metrics for the best fit model. Thereupon, in routine genetic evaluations, we suggest the use of the D model due to its computational efficiency (time of analysis required), and it was as suitable as the other models. However, as mentioned before, the current data structure might be suboptimal for maternal effect estimation. Therefore, when more data are available, the re-estimation of maternal effects for docility is recommended.

Medium-to-high heritability are in close agreement with previous heritability estimates for cattle temperament using pedigree (Burrow, 2001; Beckman et al., 2007; Haskell et al., 2014) or genomic information (Costilla et al., 2020). Therefore, yearling temperament measured on a 1–6 scale presents sufficient variability to respond to genetic selection, even though a small genetic improvement has been observed across the years in Angus cattle (Supplementary Figure S3). Finally, the maternal genetic effects contributed to 4% of the total variation in yearling temperament on the liability scale. Previous studies have also reported low maternal genetic contribution on temperament variation (Burrow, 2001; Beckman et al., 2007). Additionally, a strong negative genetic correlation was observed between additive direct and maternal genetic effects (−0.40 ± 0.039; Table 4), which can be explained by the environmental covariances between dam and offspring records and on the fixed effects’ structure used in the statistical models for the data analyses (Bijma, 2006). Additionally, the effect of sire-by-herd interaction was not included, which could potentially adjust the negative correlation (Willham, 2000). Negative genetic correlation estimates between direct and maternal additive genetic effects for other traits were also reported in the literature (Dodenhoff et al., 1999; Burrow, 2001; Bijma, 2006; Beckman et al., 2007). However, Willham (2000) stated that there is a possibility of negative correlation between maternal and direct genetic components. The aggressiveness of the dams during the nursery event could be associated with higher protectiveness of the progeny and not necessarily with overall temperament of the cow.

### Approximated Genetic Correlation Between Yearling Temperament and Economically Relevant and Indicator Traits

In general, Pearson correlation between EBVs (or EPDs) does not properly represent the genetic correlation, especially between lowly accurate values (Calo et al., 1973). Therefore, Calo et al. (1973) proposed an approach to adjust for the EBV accuracies, which has been extensively used in dairy cattle studies (Dikmen et al., 2012; Costa et al., 2020; Dechow et al., 2020). Traditionally, dairy cattle have higher EBV accuracies than beef cattle populations. Thereof, the method used by Calo et al. (1973) might penalize Pearson correlations when correlated traits have low accuracies. For this study, we calculated a correlation of EBVs weighted for the accuracies.

Pairwise genetic correlation among all other relevant traits had been performed, and similar direction and scale were observed between genetic correlation using the weighted Pearson correlation and multi-trait animal model, considering the same population (www.angus.org/Nce/Heritabilities). For completeness, we have presented the approximated genetic correlations between all pairs of traits. However, only associations between yearling temperament and the other traits are discussed in this article.

Positive and favorable genetic correlation was observed between temperament and growth, feed efficiency, precocity, and carcass traits. Similar results were observed in cattle. For instance, temperament was genetically associated with higher average daily gain, better conformation scores, finishing precocity, and muscling (Burrow, 2001; King et al., 2006; Phocas et al., 2006; Hoppe et al., 2010; Sant’Anna et al., 2012, 2019; Cooke, 2014; Coutinho et al., 2017). Nervous cattle tend to allocate more energy into the state of excitement instead of using it for other physiological functions such as growth and reproduction (Petherick et al., 2002). In terms of carcass quality, the study observed alterations in the carcass pH based on the temperament groups [i.e., more docile vs. more aggressive; (Petherick et al., 2002)]. The pH variations by animal temperament were speculated to be caused by differences in lactic acid concentrations, as nervous animals have higher levels of lactic acid as a consequence of the distress (Petherick et al., 2002). Other traits (e.g., foot score and hair shedding) had low genetic correlation with temperament, suggesting its partially independent genetic responses.

In summary, there were no unfavorable correlations between temperament and other relevant traits in livestock. This suggests that direct selection for temperament would not negatively impact the genetic improvement of other relevant traits. Actually, the long-term selection for growth, feed efficiency, precocity, and carcass traits has favorable effect on the indirect selection for temperament, or *vice versa*.

### Weighted Single-Step Genome-Wide Association Analyses

Animal temperament is a behavioral response from a multifactorial process. The starting point is a stimulus, which could be from a simple touch, sight, sound, smell, or taste, until more complex levels, for instance, an intuitive feeling of dominance. The complexity extends to the genetic level, in which behavior is controlled by many genes with small effect (Costilla et al., 2020; Alvarenga et al., 2021). Despite the high heritability estimate, our study found three pieces of evidence of a polygenic nature of yearling temperament in North American Angus. First, an even distribution of variance explained by genomic windows was observed across the genome (Figure 3). Second, none of the genomic windows explained the large proportion of the total additive genetic variance for yearling temperament—the maximum variance explained was 0.51% on BTA11 (Supplementary Table S4). Finally, at the modeling level, small differences were observed in the distribution of the genomic window effect and variance explained when using ssGWAS or WssGWAS approaches (Supplementary Figure S2). Similar findings were observed by Araujo et al. (2022) while fitting haplotype groups instead of SNP effects using the AAA dataset for temperament. In general, a weighting method (e.g., WssGWAS) is expected to prioritize major regions when a less polygenic trait is being analyzed (Wang et al., 2014). In summary, the weighting process for genomic associations using SNPs is not necessary if the target trait underlies a polygenic architecture.

At the genomic level, another characteristic of yearling temperament is the influence of the X-chromosome. In comparison with other chromosomes, X was underrepresented with a total of 97 SNPs (59.13 ± 45.90 Kbp is the average ± standard deviation distance between SNPs) out of 42,662 SNPs (58.18 ± 54.47), because we kept only pseudo-autosomal SNPs that probably follow a similar recombination pattern to that of the autosomal chromosomes. Regardless, a peak was captured on the X chromosome, but no annotated genes were identified within this region. However, this finding highlights the importance of the X-chromosome for behavioral traits, as reported by Alvarenga et al. (2021). In this context, Musante and Ropers (2014) have reviewed numerous X-chromosome defects linked to cognitive disorders in humans. Therefore, we encouraged further studies evaluating the impact of the X-chromosome on the target trait, including both PAR and non-PAR regions. Song et al. (2021) have evaluated few alternative approaches to account for the sex-linked chromosomes in genomic analyses, such as an interaction of sex-linked SNP and sex.

The QTL annotation within the selected genomic regions corroborates and potentially justifies the genetic correlation between temperament and growth, carcass traits, precocity, reproduction, and maternal traits (Figure 2). The overlapping regions offer insights into mechanisms that can cause the genetic correlation, such as pleiotropy, LD between trait-specific QTLs, and/or LD between QTL and marker (Gianola et al., 2015). The majority of the QTLs were annotated for milk content and yield, which goes in line with the extended list of association studies for milk traits and annotated in the animal QTL database. No straightforward relationship between behavioral traits and milk yield have been drawn (Hedlund and Lovlie, 2015). However, many studies observed that nervous cows tend to have lower milk production, justified by differential hormonal functions (Hedlund and Lovlie, 2015). One gene annotated for milking speed (i.e., *TOX*) is involved in the immune system (Aliahmad et al., 2012). So far, to the best of our knowledge, no direct connection has been made between behavioral traits and the *TOX* gene. In human studies, there are reports indicating that the immune system can be a mediator of adulthood behavior, for example, dysregulation of immune responses was associated with higher aggression (Takahashi et al., 2018). In cattle, *TOX* was reported in association with puberty in Brahman beef cattle (Fortes et al., 2012) and carcass traits in Korean Hanwoo cattle (Bhuiyan et al., 2018), and it has been identified as a signature of selection in tropical adapted crossbred cattle (Cheruiyot et al., 2018).

In beef cattle, yearling temperament is associated with the length of productive life of the animal (Oliveira et al., 2020), as it is one of the voluntary culling reasons. QTLs annotated for longevity also overlapped with regions for yearling temperament. The genes located in regions for longevity and yearling temperament are mainly associated and enriched in visual mechanisms (Chang et al., 2009). The majority of the genes are from the crystalline gamma family genes (e.g., *CRYGB*, *CRYGC*, and *CRYGD*). In behavior, visual perception is required for the reception of a stimulus, which is subsequently converted to a physiological messenger, and properly recognized as a signal (Chang et al., 2009).

Another term identified in this study which has no significance (*p* = 0.42) but is biologically important is methylation (Supplementary Table S5). Altered cattle behavior has been linked with differential methylation of DNA (Corvo et al., 2020; Littlejohn et al., 2020). Three genes were involved in this methylation process (i.e., *PDE6C*, *RBP4*, and *CRYGC*), and out of those, *PDE6C* was also enriched in the visual perception. In animal models (i.e., mice), this gene has been reported in retinol progenitor regions to be highly methylated, which could impact photoreceptor mechanisms and, consequently, the signaling cascades (Dvoriantchikova et al., 2019). In summary, this study identified genes participating in the visual mechanism, in which the eyes, in addition to being responsible for stimulus reception, have been defined as the window to the brain. For instance, pupil dilation has been associated with cognitive ability and Alzheimer’s disease (Granholm et al., 2017; Ozeri-Rotstain et al., 2020).

Another gene participating in the methylation process is *RBP4* (retinol binding protein gene), in which knockout mice for it showed impairment, reduced activity, and increased anxiety-like behavior (Buxbaum et al., 2014). The protein produced by the *RBP4* gene is the principal retinol (i.e., vitamin A) serum transporter in the human body (Reay and Cairns, 2020). Disruption of the transport, metabolism, and signaling of vitamin A metabolites have been associated with mental and behavioral disorders in humans [reviewed by Reay and Cairns (2020)]. In cattle, *RBP4* has been associated with growth traits, which might be related to insulin metabolism (Wang et al., 2010) and heifer fertility (Abdollahi-Arpanahi et al., 2019).

The region with the greatest additive genetic variance explained is located on BTA11, but no annotated gene was found in this region. However, there was one long noncoding RNA (lncRNA, ENSBTAG00000048646) located in a region explaining 0.49% of the total variation (Supplementary Table S4). Some lncRNA genes are specifically expressed in brain cells, which implies its roles in neuronal development and cognitive and behavioral regulation (Zhang et al., 2020). In cattle, this gene biotype has been associated with possible skin pigmentation (Weikard et al., 2013), meat quality (Billerey et al., 2014), and lipid metabolism and has been suggested as a possible heat-stress biomarker (Ibeagha-Awemu and Zhao, 2015). However, to the best of our knowledge, no study evaluated the impact of lncRNA on behavior in cattle.

Insulin metabolism is another pathway in which few of the genes identified in this study play an important role, for example, omega-3 Fatty Acid Receptor-1 gene (*FFAR4*) located on BTA26 (Marcotte et al., 2017). Insulin metabolism dysfunction could impact performance and affect the overall mood and cognitive ability of the individuals through dopaminergic abnormal functioning, as observed in humans (Kleinridders et al., 2015; Lyrae Silva et al., 2019). Similar to other genes discussed in this study (e.g., *TOX*), *FFAR4* receptors have also been reported as triggers to inflammatory responses (Oh et al., 2010). In general, *FFAR4* gene signals either a pathway involved in $G_{\alpha q}$ , which is linked with insulin resistance with action at the adipocyte, or β -arretin-2, which directly influences the inflammatory pathways with action at the macrophage [the complete process was reviewed and summarized by Oh and Walenta (2014)].

Finally, Alvarenga et al. (2021) systematically reviewed genes associated with farmed mammals’ behavior. Among all genes catalogued by the authors, two (i.e., *U6* and *CEP55*) genes previously reported to be associated with behavioral traits in livestock overlapped with the genes identified in this study (Alvarenga et al., 2021). Interestingly, the U6 spliceosomal RNA (*U6*) gene was previously reported to be associated with temperament (Hanna et al., 2014), maternal behavior (Michenet et al., 2016), and sucking reflex (Dreher et al., 2019) in cattle and feeding behavior (Cross et al., 2018) and adrenaline/creatine level (Terenina et al., 2013) in pigs. The *U6* gene is a noncoding RNA gene; in this study, the paralog gene identified is on BTA8 explaining 0.22% of the total genetics. The U6 snRNA is highly conserved in eukaryotes, and it is located in the spliceosome, where it orchestrates the splicing function (Didychuk et al., 2018), which seems to have an evolutionary importance in terms of organism viability. Molecular and chemistry studies might be crucial to underpin the role of uridine-rich small nuclear RNAs (i.e., *U6*) in alternative animal behavior. However, more studies are required to elucidate the roles of the genes identified in this study, including *in vitro* gene knockout and gene editing studies.

## Conclusions

Our study encompasses a large, diverse, and robust dataset from the North American Angus cattle population. Yearling temperament was shown to be heritable (0.38 ± 0.01), suggesting that genetic gains can be effectively obtained through direct genetic and genomic selection. Additionally, direct selection for yearling temperament is not expected to result in unfavorable effects on other economically important traits. Actually, the long-term selection for growth, feed efficiency, precocity, and carcass traits has a favorable effect on the indirect selection for temperament, or *vice versa*.

Sex and extrinsic factors affect animal behavior, such as age of dam, additional human–animal interaction, and birth region. Fitting maternal effects are not crucial for breeding value estimation of temperament in the US Angus population, suggesting the use of the reduced model due to its computational efficiency. Many of the genomic regions associated with yearling temperament were enriched in QTL regions linked with milking speed, longevity, and other economically important traits. Pseudo-autosomal regions of the X-chromosome seem to play a role in yearling temperament, as well as RNA gene biotypes, for example, long noncoding RNA genes. Finally, many of the genes were enriched in visual pathways, which in addition to being important for stimulus, have also been linked to cognitive abilities. The SNPs and genomic regions identified in this study can be used when designing customized SNP panels, to be further investigated in functional studies aiming to better understand the biological mechanisms influencing cattle temperament, as well as used for a biology-driven genomic prediction. In summary, the insights from our study will be useful for various practical applications and future research, including the breeding industry, farm management, and behavioral genomics studies.

## Data Availability

The data analyzed in this study are subject to the following licenses/restrictions: The data supporting the results of this article are included within the article and in its Supplementary Files. The raw data cannot be made available, as it is property of the American Angus Association (Saint Joseph, MO, United States) and this information is commercially sensitive. Requests to access these datasets should be directed to the corresponding author.
