# Supplementary material for: Genetic Modeling and Genomic Analyses of Yearling Temperament in American Angus Cattle and Its Relationship With Productive Efficiency and Resilience Traits
Source: Front Genet. 2022 Apr 4;13:794625. doi: 10.3389/fgene.2022.794625 (PMC9014094; doi:10.3389/fgene.2022.794625)
Supplement: Supplementary file 1 [file Table1.docx]

**Supplementary Table 1**. Least square means for the non-genetic factors potentially affecting yearling temperament.

| Variables | N. | Mean^1^ | SE | Test^2^ |
| --- | --- | --- | --- | --- |
|  | Age of dam | | | |
| 3 | 100185 | 1.34 | 0.011 | A |
| 4 | 46867 | 1.33 | 0.011 | A |
| 5 | 32708 | 1.35 | 0.011 | B |
| 6 | 25501 | 1.36 | 0.011 | BC |
| 7 | 19492 | 1.37 | 0.011 | BC |
| 8 | 14169 | 1.35 | 0.012 | BC |
| 9 | 10160 | 1.37 | 0.012 | BC |
| 10 | 7102 | 1.37 | 0.013 | BC |
| 11 | 4424 | 1.39 | 0.014 | CD |
| 12 | 5421 | 1.41 | 0.013 | D |
|  | Conception type | | | |
| Embryo’s transference | 38526 | 1.36 | 0.011 | A |
| Natural conception | 227503 | 1.37 | 0.010 | B |
|  | Parity type | | | |
| Single |  | 1.37 | 0.009 | A |
| Twin |  | 1.36 | 0.014 | A |
|  | Birth season | | | |
| Spring | 79530 | 1.41 | 0.011 | D |
| Summer | 26608 | 1.31 | 0.011 | A |
| Fall | 47910 | 1.35 | 0.011 | B |
| Winter | 111981 | 1.39 | 0.011 | C |
|  | Creep-feeding | | | |
| Non-creep-fed | 193361 | 1.40 | 0.010 | B |
| Creep-fed | 72668 | 1.32 | 0.011 | A |
|  | Sex | | | |
| Bull | 147671 | 1.28 | 0.010 | A |
| Steer | 3332 | 1.49 | 0.015 | C |
| Female | 115026 | 1.32 | 0.010 | B |
|  | If animal has ultrasound information | | | |
| No | 82110 | 1.38 | 0.011 | B |
| Yes | 183919 | 1.35 | 0.010 | A |
|  | If animal has feed intake information | | | |
| No | 264516 | 1.39 | 0.007 | B |
| Yes | 1513 | 1.33 | 0.017 | A |

^1^ Averaged over the levels of age of dam, conception type, parity type, birth season, creep-feeding, sex, and if the animal had ultrasound and/or feed intake information; ^2^ Pairwise contrast comparison considering an alpha of 0.05, and the p-values was adjusted by Tukey method, implemented in *lsmeans* package in R software; N.: number of animals; SE: standard error.
