# Supplementary material for: Genetic Modeling and Genomic Analyses of Yearling Temperament in American Angus Cattle and Its Relationship With Productive Efficiency and Resilience Traits
Source: Front Genet. 2022 Apr 4;13:794625. doi: 10.3389/fgene.2022.794625 (PMC9014094; doi:10.3389/fgene.2022.794625)
Supplement: Supplementary file 2 [file Table2.docx]

**Supplementary Table 2A.** Complete (co)variance components for the reduced model (D model).

| Effects | Mean | HPD interval (95%) | | Effective sample size | Median | Mode |
| --- | --- | --- | --- | --- | --- | --- |
| Contemporary group | 0.24 | 0.21 | 0.27 | 2872.10 | 0.24 | 0.24 |
| Animal effect | 0.28 | 0.27 | 0.30 | 328.70 | 0.28 | 0.28 |
| Residual | 0.21 | 0.18 | 0.24 | 3350.50 | 0.21 | 0.21 |
| Threshold | 1.50 | 1.01 | 2.15 | 5000.00 | 1.44 | 1.27 |
| Heritability | 0.39 | 0.36 | 0.41 | 950.20 | 0.39 | 0.39 |

500K iterations, 250K burn-in, and 50 thin; Mean: Posterior marginal mean; HPD: Highest probability density.

**Supplementary Table 2B.** Complete (co)variance components for the model including maternal genetic effect (DMG model).

| Effects | Mean | HPD interval (95%) | | Effective sample size | Median | Mode |
| --- | --- | --- | --- | --- | --- | --- |
| Contemporary group | 0.24 | 0.21 | 0.26 | 3467.00 | 0.24 | 0.23 |
| Animal effect | 0.32 | 0.30 | 0.35 | 111.30 | 0.32 | 0.32 |
| Direct x maternal genetic | -0.04 | -0.05 | -0.03 | 47.70 | -0.04 | -0.04 |
| Maternal genetic | 0.03 | 0.02 | 0.04 | 24.10 | 0.03 | 0.03 |
| Residual | 0.19 | 0.16 | 0.22 | 661.20 | 0.18 | 0.18 |
| Threshold | 1.50 | 1.02 | 2.11 | 4809.40 | 1.44 | 1.24 |
| Direct heritability | 0.44 | 0.41 | 0.47 | 119.6 | 0.44 | 0.45 |
| Maternal heritability | 0.04 | 0.03 | 0.05 | 26.9 | 0.04 | 0.04 |
| Total heritability | 0.38 | 0.35 | 0.40 | 451.50 | 0.38 | 0.38 |
| Correlation direct and maternal genetic | -0.40 | -0.47 | -0.32 | 53.7 | -0.40 | -0.38 |

800K iterations, 550K burn-in, and 50 thin; Mean: posterior marginal mean; HPD: Highest probability density.

**Supplementary Table 2C.** Complete (co)variance components for the model including maternal permanent environment effect (DMP model).

| Effects | Mean | HPD interval (95%) | | Effective sample size | Median | Mode |
| --- | --- | --- | --- | --- | --- | --- |
| Contemporary group | 0.24 | 0.21 | 0.26 | 2232.40 | 0.24 | 0.23 |
| Animal effect | 0.27 | 0.26 | 0.29 | 431.30 | 0.27 | 0.28 |
| Maternal permanent environment | 0.01 | 0.01 | 0.01 | 15.30 | 0.01 | 0.01 |
| Residual | 0.21 | 0.18 | 0.24 | 1999.20 | 0.20 | 0.20 |
| Threshold | 1.50 | 1.02 | 2.12 | 2611.00 | 1.43 | 1.24 |
| Heritability | 0.38 | 0.35 | 0.40 | 669.80 | 0.38 | 0.38 |

1000K iterations, 750K burn-in, and 100 thin; Mean: Posterior marginal mean; HPD: Highest probability density.
