# Supplementary material for: Genetic Modeling and Genomic Analyses of Yearling Temperament in American Angus Cattle and Its Relationship With Productive Efficiency and Resilience Traits
Source: Front Genet. 2022 Apr 4;13:794625. doi: 10.3389/fgene.2022.794625 (PMC9014094; doi:10.3389/fgene.2022.794625)
Supplement: Supplementary file 3 [file Table5.docx]

**Supplementary Table 5**. Functional annotation using DAVID based on Gene Ontology and KeyTerms.

| Category | Term | Gene name | Ensembl gene ID | % | Benjamini |
| --- | --- | --- | --- | --- | --- |
| UP_KEYWORDS | Eye lens protein | *CRYGB,*  *CRYGD,*  *CRYGC* | ENSBTAG00000021770, ENSBTAG00000015054, ENSBTAG00000014783 | 18.8 | 0.00 |
| UP_KEYWORDS | Methylation | *PDE6C,*  *RBP4,*  *CRYGC* | ENSBTAG00000000445, ENSBTAG00000000442, ENSBTAG00000014783 | 18.8 | 0.42 |
| GOTERM_BP | Visual perception (GO:0007601) | *PDE6C,*  *CRYGD,*  *CRYGC* | ENSBTAG00000000445, ENSBTAG00000015054, ENSBTAG00000014783 | 18.8 | 0.06 |
| GOTERM_MF | Structural constituent of eye lens (GO:0005212) | *CRYGB,*  *CRYGD,*  *CRYGC* | ENSBTAG00000021770, ENSBTAG00000015054, ENSBTAG00000014783 | 20.0 | 0.03 |
| GOTERM_MF | Metalloendopeptidase activity (GO:0004222) | *ADAMTS15,*  *ADAMTS8* | ENSBTAG00000016857, ENSBTAG00000010411 | 13.3 | 0.89 |
