# Supplementary material for: Genetic Modeling and Genomic Analyses of Yearling Temperament in American Angus Cattle and Its Relationship With Productive Efficiency and Resilience Traits
Source: Front Genet. 2022 Apr 4;13:794625. doi: 10.3389/fgene.2022.794625 (PMC9014094; doi:10.3389/fgene.2022.794625)
Supplement: Supplementary file 4 [file Table3.docx]

**Supplementary Table 3.** Weighted Pearson correlation among estimated breeding values for temperament and relevant traits for the beef industry.

| Trait 2 | N | EBV accuracy for temperament | | | EBV accuracy for trait 2 | | | $\boldsymbol{r}_{\boldsymbol{w}\boldsymbol{1,2}}$ | SE |
| --- | --- | --- | --- | --- | --- | --- | --- | --- | --- |
|  |  | **Mean** | **SD** | **Min** | **Mean** | **SD** | **Min** |  |  |
| CED | 150219 | 0.41 | 0.05 | 0.25 | 0.30 | 0.05 | 0.25 | 0.07 | 0.00 |
| BW | 264538 | 0.40 | 0.04 | 0.25 | 0.42 | 0.08 | 0.25 | -0.02 | 0.00 |
| WW | 264933 | 0.40 | 0.04 | 0.25 | 0.37 | 0.07 | 0.25 | 0.28 | 0.00 |
| YW | 258967 | 0.40 | 0.04 | 0.25 | 0.34 | 0.06 | 0.25 | 0.28 | 0.00 |
| RADG | 74567 | 0.41 | 0.05 | 0.25 | 0.32 | 0.04 | 0.25 | 0.17 | 0.00 |
| DMI | 74567 | 0.41 | 0.05 | 0.25 | 0.32 | 0.04 | 0.25 | 0.15 | 0.00 |
| YH | 192332 | 0.40 | 0.05 | 0.25 | 0.42 | 0.07 | 0.25 | 0.14 | 0.00 |
| SC | 168274 | 0.40 | 0.05 | 0.25 | 0.39 | 0.07 | 0.25 | 0.11 | 0.00 |
| HP | 35144 | 0.43 | 0.06 | 0.25 | 0.28 | 0.03 | 0.25 | 0.04 | 0.01 |
| CEM | 101388 | 0.41 | 0.06 | 0.25 | 0.30 | 0.04 | 0.25 | 0.05 | 0.00 |
| Milk | 129831 | 0.40 | 0.06 | 0.25 | 0.33 | 0.06 | 0.25 | 0.10 | 0.00 |
| MW | 105031 | 0.41 | 0.06 | 0.25 | 0.37 | 0.05 | 0.25 | 0.20 | 0.00 |
| MH | 99684 | 0.41 | 0.06 | 0.25 | 0.39 | 0.07 | 0.25 | 0.17 | 0.00 |
| CW | 185559 | 0.41 | 0.05 | 0.25 | 0.34 | 0.07 | 0.25 | 0.25 | 0.00 |
| Marb | 137486 | 0.41 | 0.05 | 0.25 | 0.32 | 0.06 | 0.25 | 0.07 | 0.00 |
| RE | 185552 | 0.41 | 0.05 | 0.25 | 0.32 | 0.05 | 0.25 | 0.18 | 0.00 |
| Fat | 119167 | 0.41 | 0.05 | 0.25 | 0.31 | 0.05 | 0.25 | 0.00 | 0.00 |
| Foot_Angle | 39996 | 0.42 | 0.05 | 0.25 | 0.28 | 0.03 | 0.25 | -0.01 | 0.01 |
| PAP | 26491 | 0.42 | 0.05 | 0.25 | 0.27 | 0.02 | 0.25 | -0.02 | 0.01 |
| HS | 52084 | 0.41 | 0.05 | 0.25 | 0.29 | 0.04 | 0.25 | -0.05 | 0.00 |

N: number of animals available with both EBVs (temperament and trait 2); SD: standard deviation; Min: minimum; $r_{w1,2}$: weighted Pearson correlation; SE: standard error; CED: calving ease direct; CEM: maternal calving ease; BW: birth weight; WW: weaning weight; YW: yearling weight; RADG: residual average daily gain; DMI: dry-matter intake; MILK: maternal milk; YH: yearling height; SC: scrotal circumference; CW: carcass weight; MARB: marbling score; RE: ribeye area; FAT: fat thickness; Foot_Angle: foot angle; PAP: pulmonary artery pressure; HS: hair shedding score; HP: heifer pregnancy; MW: mature weight; MH: mature height.
