# Supplementary material for: Genetic Modeling and Genomic Analyses of Yearling Temperament in American Angus Cattle and Its Relationship With Productive Efficiency and Resilience Traits
Source: Front Genet. 2022 Apr 4;13:794625. doi: 10.3389/fgene.2022.794625 (PMC9014094; doi:10.3389/fgene.2022.794625)
Supplement: Supplementary file 5 [file Table4.docx]

**Supplementary Table 4.** Top genomic windows explaining greater than 0.20% of the total additive genetic variance of yearling temperament.

|  | Genomic window | | | | | Gene | | | | |
| --- | --- | --- | --- | --- | --- | --- | --- | --- | --- | --- |
| CHR | **Start name** | **Start pos.** | **End name** | **End pos.** | **VE (%)** | **Ensembl ID** | **Name** | **Start pos.** | **End pos.** | **Biotype** |
| 2 | rs110119158 | 96181032 | rs110674596 | 96426927 | 0.21 | ENSBTAG00000007830 | *PLEKHM3* | 96101252 | 96318495 | Protein coding |
|  |  |  |  |  |  | ENSBTAG00000049856 |  | 96382638 | 96385511 | Protein coding |
|  |  |  |  |  |  | ENSBTAG00000015054 | *CRYGD* | 96395739 | 96397590 | Protein coding |
|  |  |  |  |  |  | ENSBTAG00000014783 | *CRYGC* | 96405966 | 96407968 | Protein coding |
|  |  |  |  |  |  | ENSBTAG00000021770 | *CRYGB* | 96417867 | 96420083 | Protein coding |
| 4 | rs110564527 | 16670558 | rs43709092 | 17065068 | 0.26 | ENSBTAG00000045937 |  | 16779419 | 16781333 | Protein coding |
|  |  |  |  |  |  | ENSBTAG00000054892 |  | 16779758 | 16779803 | miRNA |
|  |  |  |  |  |  | ENSBTAG00000006924 | *NXPH1* | 16889144 | 16890150 | Protein coding |
| 8 | rs41634298 | 26576536 | rs42263449 | 26696264 | 0.22 | ENSBTAG00000014103 | *SH3GL2* | 26598842 | 26826372 | Protein coding |
|  |  |  |  |  |  | ENSBTAG00000042797 | *U6* | 26615204 | 26615310 | snRNA |
| 8 | rs42866310 | 21145798 | rs42414701 | 21236045 | 0.21 |  |  |  |  |  |
| 10 | rs43644204 | 88674746 | rs41654591 | 88780846 | 0.30 | ENSBTAG00000020480 | *SPTLC2* | 88689279 | 88784551 | Protein coding |
|  |  |  |  |  |  | ENSBTAG00000049115 |  | 88714810 | 88715345 | Protein coding |
| 11 | rs110911179 | 16270189 | rs110132903 | 16405944 | 0.51 |  |  |  |  |  |
| 11 | rs110448193 | 85006812 | rs41649534 | 85223963 | 0.49 | ENSBTAG00000048646 |  | 85128330 | 85146771 | lncRNA |
| 14 | rs41724536 | 25082860 | rs109346299 | 25215941 | 0.26 |  |  |  |  |  |
| 26 | rs41659834 | 14769909 | rs41601927 | 14960555 | 0.33 | ENSBTAG00000016918 | *MYOF* | 14623780 | 14803157 | Protein coding |
|  |  |  |  |  |  | ENSBTAG00000049134 |  | 14815705 | 14854970 | Protein coding |
|  |  |  |  |  |  | ENSBTAG00000005129 | *CEP55* | 14823940 | 14845414 | Protein coding |
|  |  |  |  |  |  | ENSBTAG00000000437 | *FFAR4* | 14871804 | 14893035 | Protein coding |
|  |  |  |  |  |  | ENSBTAG00000000442 | *RBP4* | 14896080 | 14903110 | Protein coding |
|  |  |  |  |  |  | ENSBTAG00000000445 | *PDE6C* | 14911696 | 14964875 | Protein coding |
| 29 | rs110884398 | 36344787 | rs109710777 | 36569113 | 0.26 | ENSBTAG00000033237 |  | 36344150 | 36345409 | Protein coding |
|  |  |  |  |  |  | ENSBTAG00000019712 | *ST14* | 36351311 | 36389968 | Protein coding |
|  |  |  |  |  |  | ENSBTAG00000009210 | *ZBTB44* | 36408247 | 36429241 | Protein coding |
|  |  |  |  |  |  | ENSBTAG00000010411 | *ADAMTS8* | 36515554 | 36534251 | Protein coding |
|  |  |  |  |  |  | ENSBTAG00000016857 | *ADAMTS15* | 36560407 | 36584912 | Protein coding |
| X | rs42404525 | 136774379 | rs42375045 | 136987957 | 0.29 |  |  |  |  |  |

CHR: chromosome; VE: additive genetic variance explained by the sliding window expressed in %.
