# Supplementary material for: Genetic Modeling and Genomic Analyses of Yearling Temperament in American Angus Cattle and Its Relationship With Productive Efficiency and Resilience Traits
Source: Front Genet. 2022 Apr 4;13:794625. doi: 10.3389/fgene.2022.794625 (PMC9014094; doi:10.3389/fgene.2022.794625)
Supplement: Supplementary file 6 [file DataSheet1.docx]

***Supplementary Material***

**
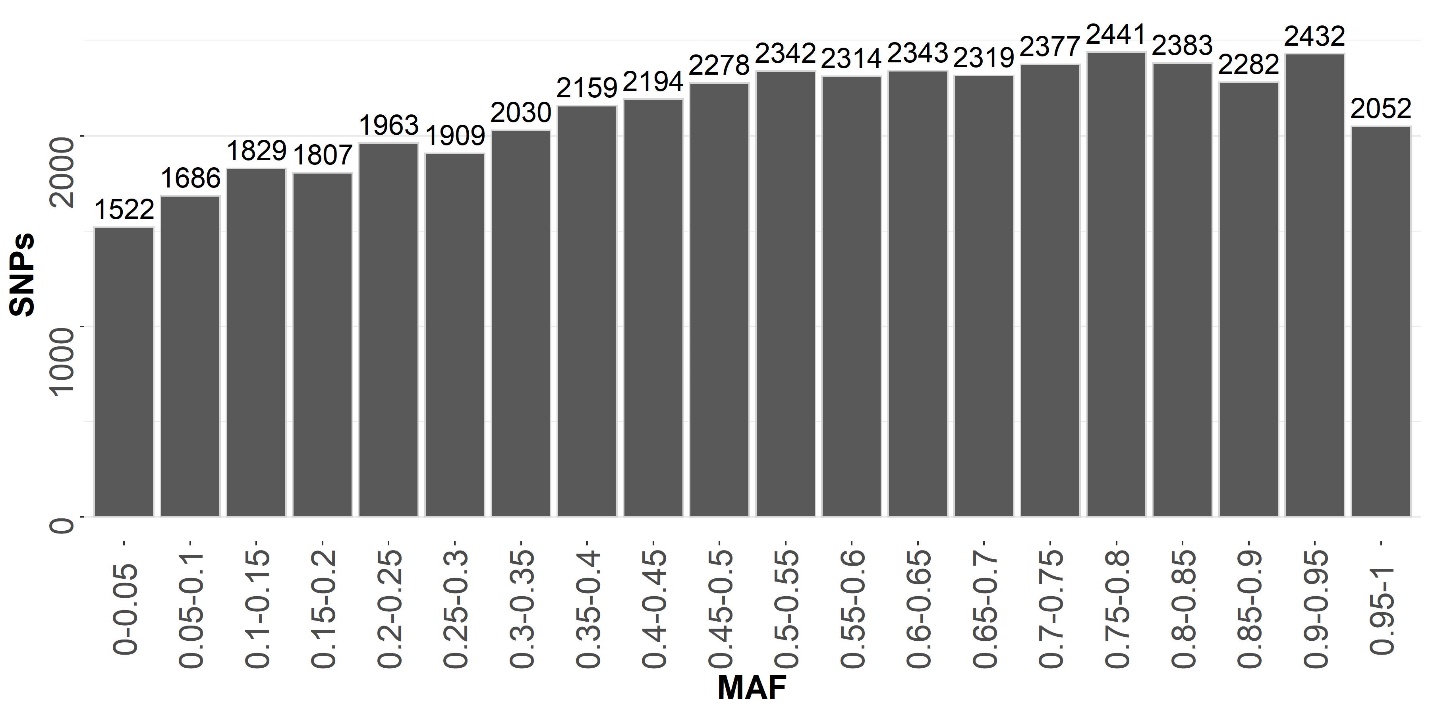
**

**Supplementary Figure 1.** Minor allele frequency distribution.

**
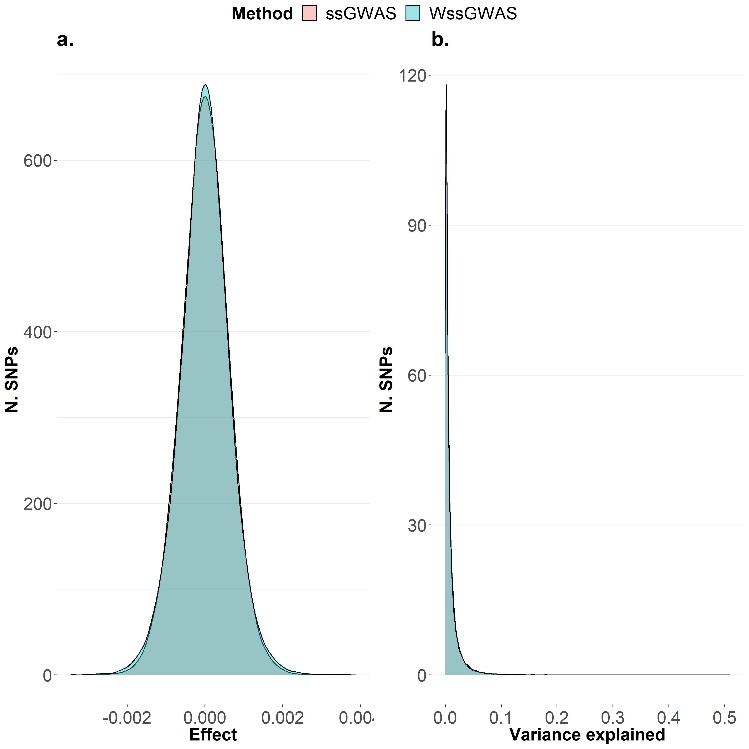
**

**Supplementary Figure 2.** Density distribution of genomic region effect and variance explained of single-step GWAS and weighted ssGWAS. a. Density distribution for the effect of each genomic region; b. density distribution for the additive genetic variance explained by each group.

**
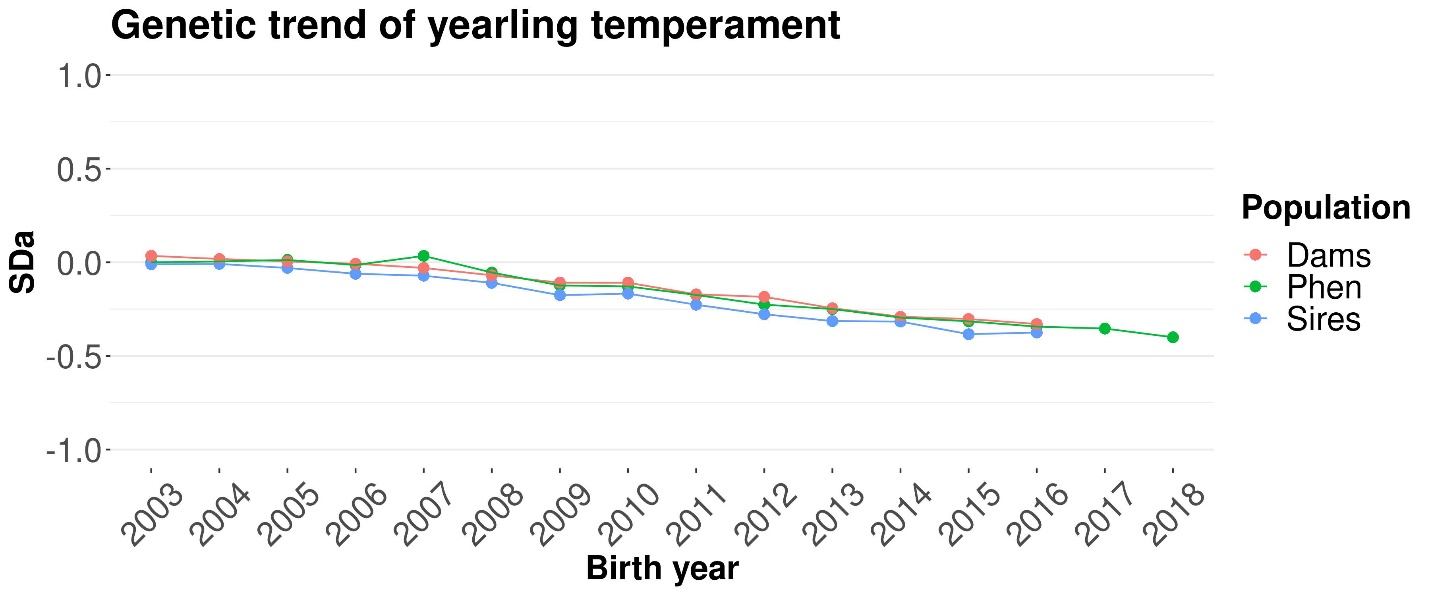
**

**Supplementary Figure 3.** Genetic trend of yearling temperament in U.S.

The genetic trends are for all Angus animals with EBVs, for sires (animals with at least one progeny in the dataset), and dams (animals with at least one progeny in the dataset). X-axis is the birth year. Y-axis is the average genetic standard deviation from the 2003 year (i.e., it was assumed as the baseline).

**Supplementary Table 1**. Least square means for the non-genetic factors potentially affecting yearling temperament.

| Variables | N. | Mean^1^ | SE | Test^2^ |
| --- | --- | --- | --- | --- |
|  | Age of dam | | | |
| 3 | 100185 | 1.34 | 0.011 | A |
| 4 | 46867 | 1.33 | 0.011 | A |
| 5 | 32708 | 1.35 | 0.011 | B |
| 6 | 25501 | 1.36 | 0.011 | BC |
| 7 | 19492 | 1.37 | 0.011 | BC |
| 8 | 14169 | 1.35 | 0.012 | BC |
| 9 | 10160 | 1.37 | 0.012 | BC |
| 10 | 7102 | 1.37 | 0.013 | BC |
| 11 | 4424 | 1.39 | 0.014 | CD |
| 12 | 5421 | 1.41 | 0.013 | D |
|  | Conception type | | | |
| Embryo’s transference | 38526 | 1.36 | 0.011 | A |
| Natural conception | 227503 | 1.37 | 0.010 | B |
|  | Parity type | | | |
| Single |  | 1.37 | 0.009 | A |
| Twin |  | 1.36 | 0.014 | A |
|  | Birth season | | | |
| Spring | 79530 | 1.41 | 0.011 | D |
| Summer | 26608 | 1.31 | 0.011 | A |
| Fall | 47910 | 1.35 | 0.011 | B |
| Winter | 111981 | 1.39 | 0.011 | C |
|  | Creep-feeding | | | |
| Non-creep-fed | 193361 | 1.40 | 0.010 | B |
| Creep-fed | 72668 | 1.32 | 0.011 | A |
|  | Sex | | | |
| Bull | 147671 | 1.28 | 0.010 | A |
| Steer | 3332 | 1.49 | 0.015 | C |
| Female | 115026 | 1.32 | 0.010 | B |
|  | If animal has ultrasound information | | | |
| No | 82110 | 1.38 | 0.011 | B |
| Yes | 183919 | 1.35 | 0.010 | A |
|  | If animal has feed intake information | | | |
| No | 264516 | 1.39 | 0.007 | B |
| Yes | 1513 | 1.33 | 0.017 | A |

^1^ Averaged over the levels of age of dam, conception type, parity type, birth season, creep-feeding, sex, and if the animal had ultrasound and/or feed intake information; ^2^ Pairwise contrast comparison considering an alpha of 0.05, and the p-values was adjusted by Tukey method, implemented in *lsmeans* package in R software; N.: number of animals; SE: standard error.

**Supplementary Table 2A.** Complete (co)variance components for the reduced model (D model).

| Effects | Mean | HPD interval (95%) | | Effective sample size | Median | Mode |
| --- | --- | --- | --- | --- | --- | --- |
| Contemporary group | 0.24 | 0.21 | 0.27 | 2872.10 | 0.24 | 0.24 |
| Animal effect | 0.28 | 0.27 | 0.30 | 328.70 | 0.28 | 0.28 |
| Residual | 0.21 | 0.18 | 0.24 | 3350.50 | 0.21 | 0.21 |
| Threshold | 1.50 | 1.01 | 2.15 | 5000.00 | 1.44 | 1.27 |
| Heritability | 0.39 | 0.36 | 0.41 | 950.20 | 0.39 | 0.39 |

500K iterations, 250K burn-in, and 50 thin; Mean: Posterior marginal mean; HPD: Highest probability density.

**Supplementary Table 2B.** Complete (co)variance components for the model including maternal genetic effect (DMG model).

| Effects | Mean | HPD interval (95%) | | Effective sample size | Median | Mode |
| --- | --- | --- | --- | --- | --- | --- |
| Contemporary group | 0.24 | 0.21 | 0.26 | 3467.00 | 0.24 | 0.23 |
| Animal effect | 0.32 | 0.30 | 0.35 | 111.30 | 0.32 | 0.32 |
| Direct x maternal genetic | -0.04 | -0.05 | -0.03 | 47.70 | -0.04 | -0.04 |
| Maternal genetic | 0.03 | 0.02 | 0.04 | 24.10 | 0.03 | 0.03 |
| Residual | 0.19 | 0.16 | 0.22 | 661.20 | 0.18 | 0.18 |
| Threshold | 1.50 | 1.02 | 2.11 | 4809.40 | 1.44 | 1.24 |
| Direct heritability | 0.38 | 0.35 | 0.40 | 451.50 | 0.38 | 0.38 |
| Maternal heritability | 0.04 | 0.03 | 0.05 | 26.9 | 0.04 | 0.04 |
| Correlation direct and maternal genetic | -0.40 | -0.47 | -0.32 | 53.7 | -0.40 | -0.38 |

800K iterations, 550K burn-in, and 50 thin; Mean: posterior marginal mean; HPD: Highest probability density.

**Supplementary Table 2C.** Complete (co)variance components for the model including maternal permanent environment effect (DMP model).

| Effects | Mean | HPD interval (95%) | | Effective sample size | Median | Mode |
| --- | --- | --- | --- | --- | --- | --- |
| Contemporary group | 0.24 | 0.21 | 0.26 | 2232.40 | 0.24 | 0.23 |
| Animal effect | 0.27 | 0.26 | 0.29 | 431.30 | 0.27 | 0.28 |
| Maternal permanent environment | 0.01 | 0.01 | 0.01 | 15.30 | 0.01 | 0.01 |
| Residual | 0.21 | 0.18 | 0.24 | 1999.20 | 0.20 | 0.20 |
| Threshold | 1.50 | 1.02 | 2.12 | 2611.00 | 1.43 | 1.24 |
| Heritability | 0.38 | 0.35 | 0.40 | 669.80 | 0.38 | 0.38 |

1000K iterations, 750K burn-in, and 100 thin; Mean: Posterior marginal mean; HPD: Highest probability density.

**Supplementary Table 3.** Weighted Pearson correlation among estimated breeding values for temperament and relevant traits for the beef industry.

| Trait 2 | N | EBV accuracy for temperament | | | EBV accuracy for trait 2 | | | $\boldsymbol{r}_{\boldsymbol{w}\boldsymbol{1,2}}$ | SE |
| --- | --- | --- | --- | --- | --- | --- | --- | --- | --- |
|  |  | **Mean** | **SD** | **Min** | **Mean** | **SD** | **Min** |  |  |
| CED | 150219 | 0.41 | 0.05 | 0.25 | 0.30 | 0.05 | 0.25 | 0.07 | 0.00 |
| BW | 264538 | 0.40 | 0.04 | 0.25 | 0.42 | 0.08 | 0.25 | -0.02 | 0.00 |
| WW | 264933 | 0.40 | 0.04 | 0.25 | 0.37 | 0.07 | 0.25 | 0.28 | 0.00 |
| YW | 258967 | 0.40 | 0.04 | 0.25 | 0.34 | 0.06 | 0.25 | 0.28 | 0.00 |
| RADG | 74567 | 0.41 | 0.05 | 0.25 | 0.32 | 0.04 | 0.25 | 0.17 | 0.00 |
| DMI | 74567 | 0.41 | 0.05 | 0.25 | 0.32 | 0.04 | 0.25 | 0.15 | 0.00 |
| YH | 192332 | 0.40 | 0.05 | 0.25 | 0.42 | 0.07 | 0.25 | 0.14 | 0.00 |
| SC | 168274 | 0.40 | 0.05 | 0.25 | 0.39 | 0.07 | 0.25 | 0.11 | 0.00 |
| HP | 35144 | 0.43 | 0.06 | 0.25 | 0.28 | 0.03 | 0.25 | 0.04 | 0.01 |
| CEM | 101388 | 0.41 | 0.06 | 0.25 | 0.30 | 0.04 | 0.25 | 0.05 | 0.00 |
| Milk | 129831 | 0.40 | 0.06 | 0.25 | 0.33 | 0.06 | 0.25 | 0.10 | 0.00 |
| MW | 105031 | 0.41 | 0.06 | 0.25 | 0.37 | 0.05 | 0.25 | 0.20 | 0.00 |
| MH | 99684 | 0.41 | 0.06 | 0.25 | 0.39 | 0.07 | 0.25 | 0.17 | 0.00 |
| CW | 185559 | 0.41 | 0.05 | 0.25 | 0.34 | 0.07 | 0.25 | 0.25 | 0.00 |
| Marb | 137486 | 0.41 | 0.05 | 0.25 | 0.32 | 0.06 | 0.25 | 0.07 | 0.00 |
| RE | 185552 | 0.41 | 0.05 | 0.25 | 0.32 | 0.05 | 0.25 | 0.18 | 0.00 |
| Fat | 119167 | 0.41 | 0.05 | 0.25 | 0.31 | 0.05 | 0.25 | 0.00 | 0.00 |
| Foot_Angle | 39996 | 0.42 | 0.05 | 0.25 | 0.28 | 0.03 | 0.25 | -0.01 | 0.01 |
| PAP | 26491 | 0.42 | 0.05 | 0.25 | 0.27 | 0.02 | 0.25 | -0.02 | 0.01 |
| HS | 52084 | 0.41 | 0.05 | 0.25 | 0.29 | 0.04 | 0.25 | -0.05 | 0.00 |

N: number of animals available with both EBVs (temperament and trait 2); SD: standard deviation; Min: minimum; $r_{w1,2}$: weighted Pearson correlation; SE: standard error; CED: calving ease direct; CEM: maternal calving ease; BW: birth weight; WW: weaning weight; YW: yearling weight; RADG: residual average daily gain; DMI: dry-matter intake; MILK: maternal milk; YH: yearling height; SC: scrotal circumference; CW: carcass weight; MARB: marbling score; RE: ribeye area; FAT: fat thickness; Foot_Angle: foot angle; PAP: pulmonary artery pressure; HS: hair shedding score; HP: heifer pregnancy; MW: mature weight; MH: mature height.

**Supplementary Table 4.** Top genomic windows explaining greater than 0.20% of the total additive genetic variance of yearling temperament.

|  | Genomic window | | | | | Gene | | | | |
| --- | --- | --- | --- | --- | --- | --- | --- | --- | --- | --- |
| CHR | **Start name** | **Start pos.** | **End name** | **End pos.** | **VE (%)** | **Ensembl ID** | **Name** | **Start pos.** | **End pos.** | **Biotype** |
| 2 | rs110119158 | 96181032 | rs110674596 | 96426927 | 0.21 | ENSBTAG00000007830 | *PLEKHM3* | 96101252 | 96318495 | Protein coding |
|  |  |  |  |  |  | ENSBTAG00000049856 |  | 96382638 | 96385511 | Protein coding |
|  |  |  |  |  |  | ENSBTAG00000015054 | *CRYGD* | 96395739 | 96397590 | Protein coding |
|  |  |  |  |  |  | ENSBTAG00000014783 | *CRYGC* | 96405966 | 96407968 | Protein coding |
|  |  |  |  |  |  | ENSBTAG00000021770 | *CRYGB* | 96417867 | 96420083 | Protein coding |
| 4 | rs110564527 | 16670558 | rs43709092 | 17065068 | 0.26 | ENSBTAG00000045937 |  | 16779419 | 16781333 | Protein coding |
|  |  |  |  |  |  | ENSBTAG00000054892 |  | 16779758 | 16779803 | miRNA |
|  |  |  |  |  |  | ENSBTAG00000006924 | *NXPH1* | 16889144 | 16890150 | Protein coding |
| 8 | rs41634298 | 26576536 | rs42263449 | 26696264 | 0.22 | ENSBTAG00000014103 | *SH3GL2* | 26598842 | 26826372 | Protein coding |
|  |  |  |  |  |  | ENSBTAG00000042797 | *U6* | 26615204 | 26615310 | snRNA |
| 8 | rs42866310 | 21145798 | rs42414701 | 21236045 | 0.21 |  |  |  |  |  |
| 10 | rs43644204 | 88674746 | rs41654591 | 88780846 | 0.30 | ENSBTAG00000020480 | *SPTLC2* | 88689279 | 88784551 | Protein coding |
|  |  |  |  |  |  | ENSBTAG00000049115 |  | 88714810 | 88715345 | Protein coding |
| 11 | rs110911179 | 16270189 | rs110132903 | 16405944 | 0.51 |  |  |  |  |  |
| 11 | rs110448193 | 85006812 | rs41649534 | 85223963 | 0.49 | ENSBTAG00000048646 |  | 85128330 | 85146771 | lncRNA |
| 14 | rs41724536 | 25082860 | rs109346299 | 25215941 | 0.26 |  |  |  |  |  |
| 26 | rs41659834 | 14769909 | rs41601927 | 14960555 | 0.33 | ENSBTAG00000016918 | *MYOF* | 14623780 | 14803157 | Protein coding |
|  |  |  |  |  |  | ENSBTAG00000049134 |  | 14815705 | 14854970 | Protein coding |
|  |  |  |  |  |  | ENSBTAG00000005129 | *CEP55* | 14823940 | 14845414 | Protein coding |
|  |  |  |  |  |  | ENSBTAG00000000437 | *FFAR4* | 14871804 | 14893035 | Protein coding |
|  |  |  |  |  |  | ENSBTAG00000000442 | *RBP4* | 14896080 | 14903110 | Protein coding |
|  |  |  |  |  |  | ENSBTAG00000000445 | *PDE6C* | 14911696 | 14964875 | Protein coding |
| 29 | rs110884398 | 36344787 | rs109710777 | 36569113 | 0.26 | ENSBTAG00000033237 |  | 36344150 | 36345409 | Protein coding |
|  |  |  |  |  |  | ENSBTAG00000019712 | *ST14* | 36351311 | 36389968 | Protein coding |
|  |  |  |  |  |  | ENSBTAG00000009210 | *ZBTB44* | 36408247 | 36429241 | Protein coding |
|  |  |  |  |  |  | ENSBTAG00000010411 | *ADAMTS8* | 36515554 | 36534251 | Protein coding |
|  |  |  |  |  |  | ENSBTAG00000016857 | *ADAMTS15* | 36560407 | 36584912 | Protein coding |
| X | rs42404525 | 136774379 | rs42375045 | 136987957 | 0.29 |  |  |  |  |  |

CHR: chromosome; VE: additive genetic variance explained by the sliding window expressed in %.

**Supplementary Table 5**. Functional annotation using DAVID based on Gene Ontology and KeyTerms.

| Category | Term | Gene name | Ensembl gene ID | % | Benjamini |
| --- | --- | --- | --- | --- | --- |
| UP_KEYWORDS | Eye lens protein | *CRYGB,*  *CRYGD,*  *CRYGC* | ENSBTAG00000021770, ENSBTAG00000015054, ENSBTAG00000014783 | 18.8 | 0.00 |
| UP_KEYWORDS | Methylation | *PDE6C,*  *RBP4,*  *CRYGC* | ENSBTAG00000000445, ENSBTAG00000000442, ENSBTAG00000014783 | 18.8 | 0.42 |
| GOTERM_BP | Visual perception (GO:0007601) | *PDE6C,*  *CRYGD,*  *CRYGC* | ENSBTAG00000000445, ENSBTAG00000015054, ENSBTAG00000014783 | 18.8 | 0.06 |
| GOTERM_MF | Structural constituent of eye lens (GO:0005212) | *CRYGB,*  *CRYGD,*  *CRYGC* | ENSBTAG00000021770, ENSBTAG00000015054, ENSBTAG00000014783 | 20.0 | 0.03 |
| GOTERM_MF | Metalloendopeptidase activity (GO:0004222) | *ADAMTS15,*  *ADAMTS8* | ENSBTAG00000016857, ENSBTAG00000010411 | 13.3 | 0.89 |
